# Opportunities for plain packaging of tobacco products in the Philippines: results of a nationwide online survey

**DOI:** 10.3389/fpubh.2024.1405062

**Published:** 2024-09-26

**Authors:** John Rafael Y. Arda, Gianna Gayle H. Amul, Eunice U. Mallari, Alen Josef A. Santiago

**Affiliations:** Ateneo Policy Center, School of Government, Ateneo de Manila University, Quezon City, Philippines

**Keywords:** plain packaging, graphic health warnings, Philippines, survey, public perception

## Abstract

**Objective:**

This study aimed to contribute to local research evidence to promote the implementation of plain packaging of tobacco products in the Philippines. The study aimed to assess Filipinos’ perception of the effectiveness of plain packaging and their readiness by assessing the potential impact of plain packaging.

**Methods:**

We conducted a nationwide geographically representative online panel survey with a sample size of 2,000 Filipinos. The survey recruited respondents 18–65 years old and residing in the Philippines, with 500 respondents each from the National Capital Region, Luzon, Visayas, and Mindanao. We showed respondents six different mock-ups of cigarette packs in plain packaging, with two sizes of graphic health warnings (50 and 75%) and three plain packaging colors (black, white, and Pantone 448C). Using five-point Likert scales, participants rated their agreement with 18 items assessing readiness and rationales for plain packaging and 54 items related to pack design (9 items for 6 pack designs).

**Results:**

The study showed that Filipinos recognize the value of adopting plain packaging with larger graphic health warning labels on tobacco products in the Philippines. Both non-smokers and smokers agreed that plain packaging has the potential to reduce the attractiveness and appeal of packs, prevent advertisement and promotion of tobacco products, reduce the ability of tobacco products to mislead consumers, increase the noticeability and effectiveness of the pictorial health warnings, increase recall of the pictorial health warnings, affect consumer perceptions of the attractiveness of the tobacco products and their relative safety, reduce youth experimentation with the use of tobacco products, prevent the use of tobacco brand variants as a promotional tool, prevent branding targeted toward youth, promote quitting among current users, and to more clearly inform consumers about the harmful effects of tobacco use.

**Conclusion:**

We recommend that policymakers pursue plain packaging as legislation or as part of a reform of the Philippines’ graphic health warnings law. The law should target tobacco products sold in the Philippines.

## 1 Introduction

The World Health Organization’s Framework Convention on Tobacco Control (WHO-FCTC) recommended using plain packaging on tobacco products (1). Plain packaging aims to decrease pack and brand appeal by prohibiting branding elements or “other features apart from health warnings, tax marks and other government-mandated information or markings (2).” Only 17 countries have implemented plain packaging; five are in Asia: Thailand, Singapore, Turkey, Saudi Arabia, and Israel (3, 4).

In the past decade, the Philippine government has shown strong political will in advancing tobacco control, particularly tobacco tax reforms (5). The Sin Tax Reform Law of 2012 increased excise taxes to PhP30 (0·54 USD) and created a single-tier tax rate for all tobacco products. The Tax Reform for Acceleration and Inclusion Law package of measures in 2017 raised tobacco taxes to PhP60 (1·08 USD) by 2023 (6). The Philippine Congress passed the Graphic Health Warnings (GHWs) Law in 2014 (7). Since the Department of Health’s first issuance of GHW templates in March 2016 (8), the law requires GHWs in all tobacco products in the Philippines, and templates are revised every 2 years (9, 10). GHW templates for vapor products, heated tobacco products, and other similar products were first issued in 2021 (11). There have been initiatives to introduce plain packaging. For example, Senate Bill No. 2191, or the “Tobacco Plain Packaging Act,” has been pending in Congress since 2019 (12). The proposed bill, which aims to require plain packaging for all tobacco products, was challenged due to a lack of local evidence in the Philippines supporting its claims that it is more effective than current GHWs (13). However, despite a significant decrease from 29.7% in 2009, smoking prevalence in the Philippines remains high at 23.8% of the adult population (1).

Research has shown that plain packaging influences the appeal of cigarette packs, reduces their attractiveness, increases awareness of smoking consequences, and influences intention to quit (14–18). Evidence from local research is crucial to policy formulation and addressing legislation and industry interference challenges (3, 19, 20). The research team’s previous study used cigarette pack mock-ups, which are scale models of a design for plain packaging (13). Our previous study presented cigarette pack mock-ups based on the GHW regulations in the Philippines and plain packaging regulations in Thailand and Singapore. We found that the mock-ups of plain packaging with larger GHWs from Thailand and Singapore were perceived to be the most effective in discouraging respondents from smoking compared to the Philippine packs with branding and smaller GHWs (13). This current study aims to build on the evidence of the potential of plain packaging in the Philippines by looking into the perceived effectiveness of plain packaging with larger GHWs and the readiness of Filipinos for plain packaging of cigarette packs.

## 2 Materials and methods

### 2.1 Design

We conducted a nationwide survey to examine (1) Filipino smokers’ perceptions of the effectiveness of plain packaging health warnings, perceived product harm and strength, and smoking-related behavior; and (2) Filipino smokers’ readiness by assessing the association of plain packaging to one’s attempt to quit, the ease of quitting, and other quitting-related cognitions. The survey recruited 2,000 respondents 18 to 65 years old and residing in the Philippines, with 500 respondents each from the National Capital Region and the country’s three major island groups - Luzon, Visayas, and Mindanao. This sample size was carefully designed to ensure equal representation of respondents from all major island groups and the most populated region. The sample size is also within the range of the sample size of national surveys conducted by Philippine Social Weather Stations and Pulse Asia (e.g., from 1,200 to 2,400 in sample size), two major public polling research organizations in the Philippines. The survey categorized respondents into four smoking profiles: smoker, occasional smoker, former smoker, and never smoker. We categorized the respondents according to smoking profiles based on questions adapted from the Global Adult Tobacco Survey and as part of the screening questions for the survey (21).

We based the survey on the research team’s previous study on GHWs (13) and relevant research from the Western Pacific region: India’s survey into the introduction of plain packaging (22); Australia’s pre-market survey about plain packaging (23); and Singapore’s Health Promotion Board’s standardized packaging study (24). Additionally, we adapted demographic questions from the Global Adult Tobacco Survey (21). The first part of the questionnaire contained demographic questions, including smoking profiles. The succeeding sections of the questionnaire showed six mock-ups of cigarette packs with plain packaging that have varying attributes (see discussion below for specific design of cigarette pack mock-up). Respondents were asked to compare and select the pack that they perceived to be the most and then the least representative of the attribute shown from the six mock-ups. (e.g., appeal, quality, perceived harm, perceived quitting attempts). We then showed each specific cigarette pack mock-up to the respondents as they answered questions about the pack and the plain packaging warning labels on a five-point Likert scale (Strongly Disagree to Strongly Agree). These packs were shown in the order of the number assigned to them in the previous section, with Pack 1 being the first and Pack 6 being the last (see Supplementary File 1 for the survey instrument).

The last part of the survey focused on smoking status and attitudes about quitting smoking, perception of plain packaging (i.e., perceived appeal, quality, harm, likeliness to smoke, effectiveness in discouraging non-users, noticeability of GHWs), and the extent to which they agree to the introduction of plain packaging in the Philippines. These questions used a five-point Likert scale and appeared in the survey as the following:

1. Overall, to what extent do you agree that this pack design is appealing to you i.e., do you like the pack/pack is attractive?
2. Overall, to what extent do you agree that this pack design encourages you to try smoking or to buy the pack?
3. Overall, to what extent do you agree that you would like to try smoking the cigarettes contained in this pack?
4. Overall, to what extent do you agree that you would like to be seen with this pack?
5. Overall, to what extent do you agree that smoking the cigarettes in this pack is harmful to your health?
6. Overall, to what extent do you agree that the health warning labels on the front of each of these packs are noticeable?
7. Overall, to what extent do you agree that the health warning labels on the front of each of these packs stand out to you/catch your attention?
8. Overall, to what extent do you agree that each of these packs makes you stop and think about the harmful effects of smoking when you look at them?
9. Overall, to what extent do you agree that the message of the health warning labels on this pack is easy to understand?

### 2.2 Mock-up cigarette packs design

**Figure 1:**
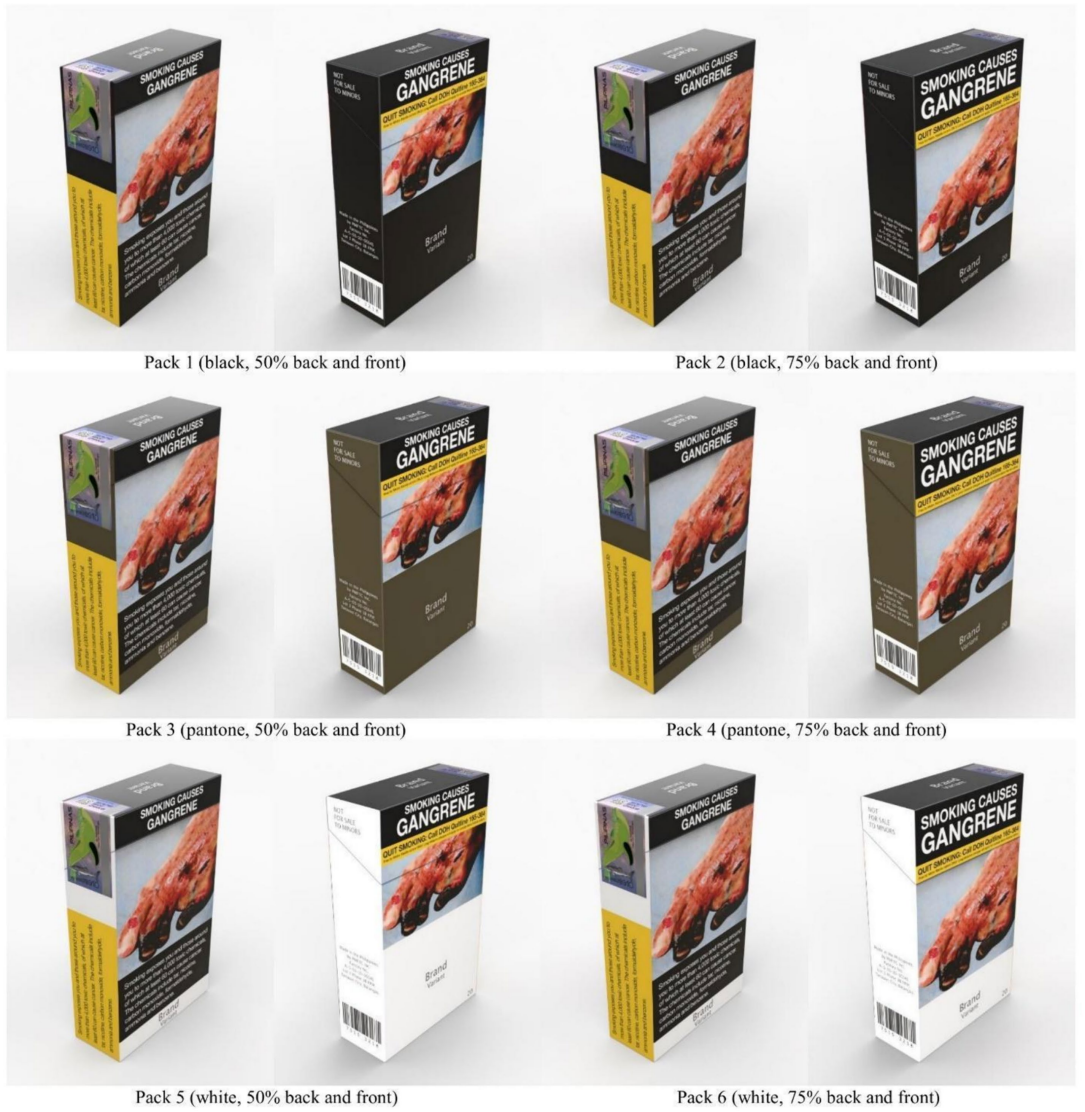
Plain packaging mock-up variations.

We designed the packs based on the authors’ previous study (13), which pointed out that Filipinos found plain packaging better at preventing smoking and making smokers quit (13). Using four main attributes of the pack that the research team found critical in a previous study—the size and location of the GHW, the visibility and location of the brand name, the colors, and the noticeability of the warnings, we designed mock-ups using popular cigarette brands and standards from the WHO and Australia’s plain packaging design as a keyframe for the plain packaging mock-ups (13). We chose one GHW (gangrene) from the Southeast Asia Tobacco Control Alliance GHW bank for all six mock-ups to eliminate perception bias on different health warnings. The packs were then designed with two sizes of GHWs (50 and 75%) and three plain packaging colors (black, white, and Pantone 448C), as shown in Figure 1.

### 2.3 Recruitment and data collection

To obtain a nationally representative sample, we contracted Rakuten Insight (Rakuten), a third-party research online survey platform. Rakuten has a panel of around 315,000 Filipinos across demographic classifications. Using this, we aimed to reach a geographical quota of 500 respondents for each region, a target gender quota, and a smoker profile quota. Rakuten then uploaded our survey instrument to their website and mobile access platform. Rakuten also shared a test link to the research team prior to the launch of the survey for design revisions and iterations to ensure user-friendliness, such as the flow and understandability of questions and compatibility of the survey design mock-up. See the implemented survey layouts for Section C and D of the survey instrument in Supplementary File 2.

Rakuten performed a soft launch as a pilot test before accepting new respondents to meet the target number of participants. There were 56 respondents in the pilot test. These responses were included in the final survey dataset, which had 2000 respondents. All the respondents in the soft launch and the official survey launch were recruited via Rakuten’s network. For both launches, Rakuten shared an invitation to the survey through their recruitment channels, including length, number of points awarded, member agreement form, and privacy policy. Individuals signed up on the platform and had to provide basic demographic and profile information to ensure they fit the panel profile. Rakuten informed registered members of our survey through email, other Rakuten invitation platforms, or their respective user profiles or home pages. The landing page of the survey shows a downloadable informed consent form, which the respondent can read before the platform shows a prompt to participate. Participation was voluntary, and the participants had the right to withdraw at any time. The respondents also had the option to respond to the questionnaire in either English or Filipino. Members who completed our survey received the number of reward points determined by Rakuten Insight that are convertible to cash payments, coupons, redeemable gifts like gift certificates, vouchers, online vouchers, and mobile phone top-ups (see Supplementary File 3 for the informed consent form).

Rakuten shared a monitoring link with the research team to show the real-time number of responses. Our survey questionnaire ran on the platform for 30 days to reach the target of 2,000 responses. While the survey did not reach the target gender and smoker profile quota, we reached the target geographic representation. Given the project’s time limitation, we decided that the geographic representation of the survey would suffice.

Rakuten generated the raw and cleaned data used for the analysis. After the survey ended, Rakuten double-checked the data manually to see whether the respondents’ demographic data fit the inclusion criteria. The research team had no access to the respondents’ personal information. This procedure is in line with existing data protection laws and protects the confidentiality and privacy of the participants.

### 2.4 Data analysis

We generated frequency and percentage distributions to describe the survey data. The variables included socio-demographic profile, smoking status and history, and perceptions of plain cigarette packaging. We used the chi-square test of independence to determine whether there is a difference between the responses of non-smokers and smokers. A statistician performed significance tests at a 5% level of significance. STATA 14 was used to generate all tables see Supplementary File 4 for the Checklist for Reporting of Survey Studies (CROSS) checklist to ensure appropriate reporting of our survey results (25).

### 2.5 Patient and public involvement

Patients or the public were not involved in the design, conduct, reporting, or dissemination plans of our research.

## 3 Results

### 3.1 Demographic profile and smoking status

The survey was open from 21 September 2022 to 25 October 2022 and recruited 2,000 respondents. Our respondents included 985 males, 985 females, and 30 gender-diverse respondents. Most respondents fell within the 18-24-year-old range (36·9%), came from the NCR (25%), finished college (55·8%), worked for a non-government or private organization (41·3%), and had an income between PHP 8,500 to PHP 39,999 (55·8%) (see Supplementary File 5 for the demographic profile of the respondents). Among respondents, 28·9% were never smokers or non-smokers, 26·9% were former smokers, 25·5% were daily smokers, and 18·9% were occasional smokers (see Supplementary File 6 for the smoker status of the respondents).

#### 3.1.1 Daily smokers

Among daily smokers (*N* = 509), 69·7% were male, 29·7% were female, and 0·6% were gender diverse. The majority (66%) fell under the 24–34 (35·2%) and the 35–44 age groups (30·8%). Among the smokers, 59·7% bought cigarettes by the pack. Among daily smokers, 57·6% were dual users: those who smoked and used e-cigarettes.

#### 3.1.2 Occasional smokers

Among occasional smokers (*N* = 377), 52·5% were male, 44·0% were female, and 3·4% were gender diverse. The majority came from the 24–34 age group (32·4%). Most occasional smokers (62·9%) buy cigarettes by the stick. At least half of the occasional smokers (50·9%) use e-cigarettes.

#### 3.1.3 Never-smokers and former smokers

Among never-smokers (*N* = 577), the majority are female (62·9%), 34·8% are male, and 2·3% are gender-diverse. Former smokers (*N* = 537) comprised 56·8% males, 43·0% females, and 0·2% gender diverse. Most never-smokers (67·6%) and former smokers (36.5%) are from the 18–24 age group. Among the non-smokers (*N* = 1,114), 10·1% have recently quit smoking with e-cigarettes, and 5·7% have never smoked a cigarette but use e-cigarettes. At least 49·4% of non-smokers are never smokers or those who have never smoked a cigarette (see Supplementary File 7 for details).

### 3.2 Potential of introducing plain packaging in the Philippines

Both non-smokers and smokers agreed that plain packaging has the potential to reduce the attractiveness and appeal of packs (64.7 and 71.3%), prevent advertisement and promotion of tobacco products (66.7 and 71.5%), reduce the ability of tobacco products to mislead consumers (65.1 and 73.4%), increase the noticeability and effectiveness of the pictorial health warnings (72.3 and 75.5%), increase recall of the pictorial health warnings (72.8 and 78%), affect consumer perceptions of the attractiveness of the tobacco products (67.6 and 74.9%) and their relative safety (67.7 and 73.1%), reduce youth experimentation with the use of tobacco products (64.8 and 72.8%), prevent the use of tobacco brand variants as a promotional tool (65.8 and 71.6%), prevent branding and glamorisation primarily targeted toward youth (68.4 and 74.4%), promote quitting among current users (65.2 and 68.1%), and to more clearly inform consumers about the harmful effects of tobacco use (76 and 80.2%).

The findings on attractiveness, brand variants misleading consumers, increased recall, consumers’ perceptions of tobacco attractiveness, reduction of youth experimentation, prevent youth-targeted branding and glamorisation, and informing consumers about harmful effects more clearly all have significant associations with the smoking status of the respondent (*p* < 0.05) (see Table 1 for more details).

**Table 1:** Perceptions to rationale for a plain packaging policy.

Attributes	Strongly agree				Agree				Neutral				Disagree				Strongly disagree				*p*-value
	Smoker		Non-smoker		Smoker		Non-smoker		Smoker		Non-smoker		Smoker		Non-smoker		Smoker		Non-smoker		
	#	%	#	%	#	%	#	%	#	%	#	%	#	%	#	%	#	%	#	%	
Reduce the attractiveness and appeal	276	31.2%	320	28.7%	355	40.1%	401	36.0%	148	16.7%	227	20.4%	85	9.6%	120	10.8%	22	2.5%	46	4.1%	0.023
Prevent advertisement and promotion on tobacco products packages	269	30.4%	336	30.2%	364	41.1%	407	36.5%	161	18.2%	255	22.9%	71	8.0%	81	7.3%	21	2.4%	35	3.1%	0.053
Reduce ability of tobacco products packages to mislead consumers (with brand variants such as mild, smooth, lights)	269	30.4%	320	28.7%	381	43.0%	405	36.4%	164	18.5%	272	24.4%	55	6.2%	86	7.7%	17	1.9%	31	2.8%	0.002
Increase the noticeability and effectiveness of the pictorial health warnings	317	35.8%	425	38.2%	352	39.7%	380	34.1%	144	16.3%	212	19.0%	58	6.5%	71	6.4%	15	1.7%	26	2.3%	0.086
Increase recall of the pictorial health warnings	324	36.6%	417	37.4%	367	41.4%	394	35.4%	135	15.2%	214	19.2%	47	5.3%	60	5.4%	13	1.5%	29	2.6%	0.015
Affect consumer perceptions of the attractiveness of the tobacco products	281	31.7%	323	29.0%	383	43.2%	430	38.6%	172	19.4%	265	23.8%	38	4.3%	68	6.1%	12	1.4%	28	2.5%	0.006
Affect consumer perceptions of the relative safety of the tobacco products	282	31.8%	324	29.1%	366	41.3%	430	38.6%	180	20.3%	266	23.9%	47	5.3%	69	6.2%	11	1.2%	25	2.2%	0.079
Reduce youth experimentation with use of tobacco products	285	32.2%	353	31.7%	360	40.6%	369	33.1%	173	19.5%	278	25.0%	51	5.8%	80	7.2%	17	1.9%	34	3.1%	0.001
Prevent the use of brand variants (lights, mild, smooth) of tobacco products as a promotional tool	272	30.7%	298	26.8%	362	40.9%	435	39.0%	186	21.0%	286	25.7%	48	5.4%	68	6.1%	18	2.0%	27	2.4%	0.077
Prevent branding and glamorization especially targeted toward youth (specific colors, design and descriptors like jazz, cool)	299	33.7%	345	31.0%	361	40.7%	417	37.4%	179	20.2%	261	23.4%	37	4.2%	62	5.6%	10	1.1%	29	2.6%	0.015
Promote quitting among current users	274	30.9%	379	34.0%	330	37.2%	348	31.2%	203	22.9%	270	24.2%	57	6.4%	86	7.7%	22	2.5%	31	2.8%	0.079
More clearly inform consumers about the harmful effects of tobacco use	350	39.5%	472	42.4%	361	40.7%	374	33.6%	135	15.2%	198	17.8%	29	3.3%	46	4.1%	11	1.2%	24	2.2%	0.011

### 3.3 Perception of plain packaging and health warnings

Both non-smokers and smokers agreed that tobacco products should come in plain packaging (72·7% and 62·9%), that they agree with the proposal (69·6% and 63·6%), that current health warnings should be larger (76·2% and 74·8%), that plain packaging can decrease tobacco use (56·5% and 51·5%), that plain packaging is relevant to the Philippine context (64% and 54·8%), and that it is possible to adopt plain packaging in the Philippines (69·6% and 59·5%). All questions except the third one - To what extent do you agree that current health warning labels on tobacco packaging in the Philippines should be larger than what it currently is?—has a significant relationship with smoking status (*p* < 0.05) (see details in Table 2).

**Table 2:** Perceptions to plain packaging and health warnings.

	Type of Respondent	Strongly Agree		Agree		Neutral		Disagree		Strongly Disagree		*p*-value
		*n*	%	*n*	%	*n*	%	*n*	%	*n*	%	
To what extent do you agree that tobacco products (e.g., cigarettes, cigars, roll-your-own tobacco, e-cigarettes, vape products, etc.) should come in standardized plain packaging?	Smoker	333	37.6%	311	35.1%	153	17.3%	65	7.3%	24	2.7%	<0.001
	Non-smoker	375	33.7%	325	29.2%	245	22.0%	102	9.2%	67	6.0%	
Overall, to what extent do you agree or disagree with this standardized plain packaging proposal?	Smoker	281	31.7%	336	37.9%	156	17.6%	82	9.3%	31	3.5%	<0.001
	Non-smoker	315	28.3%	393	35.3%	234	21.0%	128	11.5%	44	3.9%	
To what extent do you agree that current health warning labels on tobacco packaging (e.g., cigarettes, cigars, etc.) of the Philippines should be larger than what it currently is?	Smoker	344	38.8%	331	37.4%	149	16.8%	50	5.6%	12	1.4%	0.119
	Non-smoker	478	42.9%	355	31.9%	200	18.0%	61	5.5%	20	1.8%	
To what extent do you agree that plain packaging of tobacco products can decrease tobacco use?	Smoker	218	24.6%	283	31.9%	225	25.4%	134	15.1%	26	2.9%	0.007
	Non-smoker	236	21.2%	338	30.3%	331	29.7%	149	13.4%	60	5.4%	
To what extent do you agree that plain packaging is relevant to the Philippine context?	Smoker	238	26.9%	329	37.1%	229	25.8%	69	7.8%	21	2.4%	<0.001
	Non-smoker	259	23.2%	352	31.6%	329	29.5%	119	10.7%	55	4.9%	
To what extent do you agree it is possible to adopt plain packaging in the Philippines?	Smoker	259	29.2%	358	40.4%	187	21.1%	65	7.3%	17	1.9%	<0.001
	Non-smoker	278	25.0%	384	34.5%	304	27.3%	95	8.5%	53	4.8%	

### 3.4 Smokers’ and non-smokers’ perceptions of the six packs

Among the six mock-up cigarette packs, both non-smokers and smokers ranked Pack 1 as the most visually appealing pack overall, the pack with the highest quality cigarettes, the most harmful to health, simultaneously the easiest and hardest to quit with, and the pack they would not smoke. Both also agreed that Pack 6 was the pack with the lowest quality cigarettes, the least harmful to health, and the least effective pack, and that Pack 2 was the most effective pack. Non-smokers’ and smokers’ top ranked packs only differed when asked which was the least visually appealing overall (Pack 5 vs. Pack 6) and which pack they would smoke (Pack 6 vs. Pack 1) (see details in Table 3). For specific ranking percentages, see Supplementary File 8.

**Table 3:** Comparison of pack attributes among smokers and non-smokers.

Attributes	Type of Respondent	1st	2nd	3rd	4th	5th	6th
Most visually appealing overall	Smoker	P1	P2	P6	P3	P5	P4
	Non-smoker	P1	P2	P6	P3	P4	P5
Least visually appealing overall	Smoker	P6	P1	P5	P2	P3	P4
	Non-smoker	P5	P6	P1	P3	P2	P4
Highest quality cigarettes	Smoker	P1	P2	P6	P3	P5	P4
	Non-smoker	P1	P2	P6	P3	P4	P5
Lowest quality cigarettes	Smoker	P6	P5	P1	P4	P3	P2
	Non-smoker	P6	P5	P1	P3	P4	P2
Most harmful to health	Smoker	P1	P2	P6	P4	P3	P5
	Non-smoker	P1	P2	P6	P4	P3	P5
Least harmful to health	Smoker	P6	P5	P1	P3	P4	P2
	Non-smoker	P6	P5	P1	P3	P4	P2
Easiest to quit	Smoker	P1	P6	P2	P5	P3	P4
	Non-smoker	P1 and P6		P5	P2	P4	P3
Hardest to quit	Smoker	P1	P2	P6	P5	P4	P3
	Non-smoker	P1	P2	P6	P5	P4	P3
I would not smoke this	Smoker	P1	P6	P2	P3	P4	P5
	Non-smoker	P1	P6	P2	P3	P4	P5
I would smoke this	Smoker	P1	P6	P5	P2	P3	P4
	Non-smoker	P6	P1	P2	P5	P3	P4
Most effective	Smoker	P2	P1	P6	P4	P3	P5
	Non-smoker	P2	P1	P6	P5	P4	P3
Least effective	Smoker	P6	P5	P1	P3	P2	P4
	Non-smoker	P6	P5	P1	P2	P3	P4

For respondents’ perceptions of individual packs, both non-smokers agreed that Pack 1, 2, 5, and 6 were appealing to them, that the pack design encourages them to try smoking or buy the pack, that they would like to try smoking the cigarettes in the pack, that they would like to be seen with the pack, that smoking the cigarettes in the pack is harmful to their health, that the health warning labels on the front of the packs are noticeable, that the health warning labels stand out or catch their attention, that the packs make them stop and think about the harmful effects of smoking, and that the health warning labels are easy to understand. However, while smokers agreed that packs 3 and 4 are representative of these attributes as well, non-smokers did not share the same perception of the packs in terms of encouraging them to try smoking or buying the pack, of wanting to try smoking the packs’ cigarettes, and of liking being seen with the pack. For more details on these perceptions, see Supplementary File 9.

## 4 Discussion

This study contributes to local evidence on the potential implementation of plain packaging of tobacco products in the Philippines, particularly toward supporting its legislation. Most smokers and non-smokers agreed with the proposal to introduce plain packaging on current health warning labels of tobacco products. Both non-smokers and smokers perceived that plain packaging potentially reduces the attractiveness and appeal of tobacco products, encourages quitting among current smokers and informs consumers about the harmful effects of tobacco use. As for the pack mock-ups, smokers and non-smokers perceived Pack 2 as the most effective in discouraging respondents from smoking. They perceived Pack 1 as the most visually appealing, with the highest quality cigarettes, and the most harmful to health.

The current research contributes to the literature on perception on plain packaging in the Philippines. The main findings of this study aligned with our previous research on graphic health warnings and plain packaging in the Philippines (13). In the previous study, most of the respondents agreed that plain packaging in tobacco products, with larger GHWs and less branding, is better at discouraging smoking.

The design of the plain packaging mock-ups used in this study aligns with Articles 11 and 13 of the WHO-FCTC and their Guidelines for Implementation and the key policy objectives for a plain packaging law. The result of our study is also consistent with our initial research that mock-up packs with GHWs designed based on Philippine regulations are ineffective compared to the plain packaging of Singapore and Thailand packs (13). This finding is consistent with research on the effects of larger GHWs and plain packaging in Australia (26), in India (16), and their impact on adolescents and young adult smokers (14). While Australia’s plain packaging law was successful in justifying plain packaging in domestic courts and international tribunals on public health grounds, it is critical to note that the tobacco industry can still interfere in the introduction of the measure in a low-and middle-income country such as the Philippines at all stages of the policy process, whether in terms of delaying or watering down the measure (27).

Moreover, it is worth noting that given the increase in e-cigarette use in the Philippines (28), the country’s Department of Health should also explore supporting research on the feasibility and acceptability of plain packaging of e-cigarettes.

### 4.1 Study limitations

We recognize the limitation in our study’s design as we conducted a nationwide online survey through the Rakuten Insights platform, including a panel of around 315,000 Filipinos. Because of the nature of online surveys, the sample skews toward younger respondents with higher education and access to the internet and social media. Despite this limitation, we ensured geographical representation and representation across smoking profiles. While this can be considered a limitation, it can also be considered an advantage since the survey reached the youth (15–30 years of age) comprising about 30% of the Philippines’ population (29). We also recognize that there might be possible bias in the lack of randomization of our packs’ presentation when evaluating the respondents’ perceptions of each plain packaging mock-up—studies have noted that the order of elements in a Likert scale-style questionnaire may impact survey responses (30).

It is also possible that respondents may have limited exposure to and familiarity with cigarettes in plain packaging. Since we only showed the back and front of the packs, the two-dimensional digital image may limit their overall perception of the pack. This study as a baseline can benefit further evaluations of the GHW law and a potential plain packaging law (13).

Finally, while some respondents reported e-cigarette use, we only asked about respondents’ perceptions of the plain packaging of cigarettes. With the increasing number of e-cigarette users in the Philippines, tobacco control researchers in the Philippines and other low- and middle-income countries where e-cigarettes are legally sold and available in the market should include assessing the impact of health warnings and plain packaging of e-cigarette products in their research agenda.

### 4.2 Conclusion and recommendations

The survey shows that Filipinos are ready for plain packaging of tobacco products. Policymakers should tap into this evidence for an opportunity to uphold the Filipinos’ right to health and to help fulfill the Philippines’ obligations to the FCTC by introducing plain packaging on tobacco products - with larger, more effective, and noticeable graphic health warnings and less attractive and less appealing packaging, devoid of misleading information. Local and international tobacco control civil society organizations should provide technical and legal support for proponents of a plain packaging policy in low- and middle-income countries like the Philippines to avoid tobacco industry interference in the policy process, whether through lobbying or litigation.

## Data Availability

The datasets presented in this article are not readily available because of restrictions from data privacy laws and the approved research protocol for the study. Requests to access the datasets should be directed to jarda@ateneo.edu.

## References

[1] World Health Organization. Tobacco plain packaging: Global status 2021 update. Geneva: World Health Organization (2022).

[2] World Health Organization. (2008) Third session of the conference of the parties to the WHO FCTC. Geneva. Available at: http://www.who.int/fctc/cop/sessions/third_session_cop/en/.

[3] Cohen JE Zhou S Goodchild M Allwright S. Plain packaging of tobacco products: lessons for the next round of implementing countries. Tob Induc Dis. (2020) 18:1–3. doi: 10.18332/tid/130378, PMID: 33209102 PMC7670849

[4] Jetly K Mohammed Nawi A Mohd Ghazali Q Abd Manaf MR. Plain packaging and pictorial warning in Asia countries: where are we? Int J Public Health Res. (2022) 12:1545–55. doi: 10.17576/ijphr.1201.2022.08

[5] Amul GGH Etter JF. Comparing tobacco and alcohol policies from a health systems perspective: the cases of the Philippines and Singapore. Int J Public Health. (2022) 67:1605050. doi: 10.3389/ijph.2022.1605050 36312317 PMC9606809

[6] Republic of the Philippines (2017). Republic Act No. 10963: Train Reform for Acceleration and Inclusion (TRAIN). Available at: https://taxreform.dof.gov.ph/tax-reform-packages/p1-train/. Accessed on 2022.

[7] Republic of the Philippines (2014). Republic Act No. 10643: An Act to Effectively Instill Health Consciousness Through Graphic Health Warnings on Tobacco Products. Available at: https://www.officialgazette.gov.ph/2014/07/15/republic-act-no-10643/. [Accessed 2022].

[8] Administrative Order No. 0047. (2014). Department of Health (DOH). Templates and Guidelines on the Use of Templates of Graphic Health Warnings Pursuant to Republic Act No 10643. Available at: https://assets.tobaccocontrollaws.org/uploads/legislation/Philippines/Philippines-Order-No.-2014-0037-on-GHW-Template-national.pdf.

[9] Dorotheo U. (2018). The Long Road to graphic health warnings in the Philippines. Framework Convention Alliance. Available at: https://fctc.org/the-long-road-to-graphic-health-warnings-in-the-philippines/. Accessed on 27 April 2023.

[10] SEATCA. Tobacco companies must comply with new graphic health warnings by 3 march. Southeast: Asia Tobacco Control Alliance (SEATCA) (2018).

[11] Department of Health. (2021), DOH releases the first set of graphic health warning templates on Vapor Products, Heated Tobacco Products (HTPs), And Other Similar Products. Available at: https://doh.gov.ph/press-release/DOH-RELEASES-THE-FIRST-SET-OF-GRAPHIC-HEALTH-WARNING-TEMPLATES-ON-VAPOR%20PRODUCTS-HEATED-TOBACCO-PRODUCTS-%28HTPs%29-AND-OTHER-SIMILAR-PRODUCTS. Accessed on 27 April 2023.

[12] Senate of the Philippines. (2019). Tobacco Plain Packaging Act or An Act requiring the use of Plain Packaging for all Tobacco Products. Available at: https://legacy.senate.gov.ph/lis/bill_res.aspx?congress=17&q=SBN-2191.

[13] Amul GG Mallari EU Arda JRY Santiago AJA. Graphic health warnings and plain packaging in the Philippines: results of online and household surveys. Front Public Health. (2023) 11:1207779. doi: 10.3389/fpubh.2023.1207779, PMID: 37822542 PMC10562603

[14] Johnson AC Luta G Tercyak KP Niaura RS Mays D. Effects of pictorial warning label message framing and standardized packaging on cigarette packaging appeal among young adult smokers. Addict Behav. (2021) 120:106951. doi: 10.1016/j.addbeh.2021.106951, PMID: 33895661 PMC8184603

[15] Drovandi A Teague PA Glass B Malau-Aduli B. A systematic review of the perceptions of adolescents on graphic health warnings and plain packaging of cigarettes. Syst Rev. (2019) 8:25:25–15. doi: 10.1186/s13643-018-0933-0, PMID: 30654833 PMC6335796

[16] Gallopel-Morvan K Moodie C Guignard R Eker F Béguinot E. Consumer perceptions of cigarette design in France: a comparison of regular, slim, pink and plain cigarettes. Nicotine Tob Res. (2019) 21:911–7. doi: 10.1093/ntr/nty105, PMID: 29800331

[17] Nazar GP Arora M Gupta VK Rawal T Yadav A Kannuri NK. Adolescent and adult perceptions of the effects of larger size graphic health warnings on conventional and plain tobacco packs in India: a community-based cross-sectional study. Tob Induc Dis. (2019) 17:70. doi: 10.18332/tid/110677, PMID: 31636525 PMC6786002

[18] Lilic N Stretton M Prakash M. How effective is the plain packaging of tobacco policy on rates of intention to quit smoking and changing attitudes to smoking? ANZ J Surg. (2018) 88:825–30. doi: 10.1111/ans.14679, PMID: 29873162

[19] Moodie C Hoek J Hammond D Gallopel-Morvan K Sendoya D Rosen L. Plain tobacco packaging: progress, challenges, learning and opportunities. Tob Control. (2022) 31:263–71. doi: 10.1136/tobaccocontrol-2021-056559, PMID: 35241599

[20] Hughes N Arora M Grills N. Perceptions and impact of plain packaging of tobacco products in low and middle income countries, middle to upper income countries and low-income settings in high-income countries: a systematic review of the literature. BMJ Open. (2016) 6:e010391. doi: 10.1136/bmjopen-2015-010391, PMID: 27000787 PMC4809104

[21] Asma S Mackay J Song SY Zhao SY Morton J Palipudi KM. Global adult tobacco survey (GATS). Tobacco Free Initiative (TFI) (2015). World Health Organization.

[22] Arora M Tewari A Grills N Nazar GP Sonrexa J Gupta VK. Exploring perception of Indians about plain packaging of tobacco products: a mixed method research. Front Public Health. (2013) 1:35. doi: 10.3389/fpubh.2013.00035, PMID: 24350204 PMC3859976

[23] Moon G. B. (2011). Market research to determine effective plain packaging of tobacco products Canberra: Department of Health and Ageing. Available at: https://www.health.gov.au/sites/default/files/market-research-to-determine-effective-plain-packaging-of-tobacco-products.pdf.

[24] Singapore Health Promotion Board (2016). Findings for Tobacco Packaging Study (Quantitative). Available at: https://www.moh.gov.sg/docs/librariesprovider5/default-document-library/consulting-group---asia-insight-findings-for-tobacco-packaging-study-(quantitative)-health-promotion-board-singapore-2016_.pdf.

[25] Sharma A Minh Duc NT Luu Lam Thang T Nam NH Ng SJ Abbas KS. A consensus-based checklist for reporting of survey studies (CROSS). J Gen Intern Med. (2021) 36:3179–87. doi: 10.1007/s11606-021-06737-1, PMID: 33886027 PMC8481359

[26] Underwood D Sun S Welters RA. The effectiveness of plain packaging in discouraging tobacco consumption in Australia. Nat Hum Behav. (2020) 4:1273–84. doi: 10.1038/s41562-020-00940-6, PMID: 32958901

[27] van der Eijk Y Yang AY. Tobacco industry marketing adaptations to Singapore plain packaging. Tob Control. (2022) 31:744–9. doi: 10.1136/tobaccocontrol-2020-056324 33980723

[28] Sese LV Guillermo MCL. E-smoking out the facts: the Philippines’ vaping dilemma. Tobacco Use Insights. (2023) 16:1179173X2311722. doi: 10.1177/1179173x231172259, PMID: 37114161 PMC10126635

[29] Philippine Statistics Authority (2022). Age and Sex Distribution in the Philippine Population (2020 Census of Population and Housing). Available at: https://psa.gov.ph/content/age-and-sex-distribution-philippine-population-2020-census-population-and-housing

[30] Hitczenko M. (2013). Modeling anchoring effects in sequential likert scale questions. Working paper series, Federal Reserve Bank of Boston. Available at: https://hdl.handle.net/10419/107236 [Accessed on 5 August 2024].

