# Supplementary material for: Opportunities for plain packaging of tobacco products in the Philippines: results of a nationwide online survey
Source: Front Public Health. 2024 Sep 26;12:1405062. doi: 10.3389/fpubh.2024.1405062 (PMC11464346; doi:10.3389/fpubh.2024.1405062)
Supplement: Supplementary file 1 [file Data_Sheet_1.docx]

**Supplementary File 1. Survey Instrument**

***page 1* TITLE HEADER:**

# What do Filipinos think of standardized plain packaging of tobacco products or cigarette packs?

***page 1***

Are you a Filipino and aged 18 to 65 years old? If you are, then you can help us find out! We are researchers from Ateneo de Manila University and our survey is about how Filipinos perceive the implementation of standardized plain packaging in the Philippines, its comparison to a fully branded packaging, and how plain packaging impacts smoking behaviors and quitting related cognitions. The results of this policy research will support the Department of Health and legislators in advancing policy discussion and shaping policies for introducing plain packaging and other tobacco control measures.

To take part, here’s what you need to know:

- It takes about 15 to 20 minutes.
- It’s completely voluntary.
- You don't have to answer any question you don’t want to and you can stop at any time.
- Your information and your survey responses will be anonymous to the researchers.
- The survey responses will be stored in secure facilities, accessible to research team members only.

For more information: You can email [policycenter.asog@ateneo.edu](mailto:policycenter.asog@ateneo.edu) with any questions about the research.

Thinking of quitting? For questions about your own smoking habit, you could talk to your doctor or reach out to the Department of Health’s quitline: 165-364 or SMS 09212039534 or 09776277539.

For those who may need psychological counseling, we can refer them to the Ateneo Bulatao Center ([bulataocenter.ls@ateneo.edu](mailto:bulataocenter.ls@ateneo.edu)), UGAT Foundation (<https://www.facebook.com/ugatfoundationinc/>), UGAT SandaLine (<https://www.facebook.com/UGATSandaLinePage/>), or the Philippine Mental Health Association. You may also refer to the following contact details of various organizations that provide mental health care services through this link: <https://bit.ly/2TZ9cDc>.

This project has been reviewed by the Ateneo de Manila University’s Research Ethics Committee (UREO). Mr Alen Josef Santiago, Ms Gianna Gayle Amul, Ms Eunice Mallari, and Mr. John Rafael Arda are responsible for the ethical conduct of this research.

If you have any concerns about the conduct of this research that you want to raise with someone other than the researchers, please contact Ateneo IRB at (+63) 945 213 6758.

By proceeding to the survey, you voluntarily agree to participate, you confirm that you're

18 years old and above, currently residing in the Philippines, and that you are not employed or have been employed by the tobacco industry.

You may read and download the full consent form via this link: https://bit.ly/3p69XxL

***page 1* insert JOIN THE SURVEY button**

# *page 2*

**SECTION A: Demographics**

We would first like to know your background.

1. Gender
   1. Male
   2. Female
   3. Gender diverse or LGBTQIA (lesbian, gay, bisexual, transgender, queer (or those questioning their gender identity or sexual orientation, intersex, and asexual or their allies)
2. Age
   1. How old are you? (18 to 65 years old only)
3. Your location (according to region) (Select from dropdown menu)
   1. National Capital Region
   2. Cordillera Administrative Region
   3. Region I (Ilocos Region)
   4. Region II (Cagayan Valley)
   5. Region III (Central Luzon)
   6. Region IV-A (CALABARZON)
   7. Region IV-B (MIMAROPA)
   8. Region V (Bicol Region)
   9. Region VI (Western Visayas)
   10. Region VII (Central Visayas)
   11. Region VIII (Eastern Visayas)
   12. Region IX (Zamboanga Peninsula)
   13. Region X (Northern Mindanao)
   14. Region XI (Davao Region)
   15. Region XII (Soccsksargen)
   16. Region XIII (CARAGA)
   17. ARMM
4. What is the highest level of education you have completed? (Select only one)
   1. No grade completed
   2. Preschool
   3. Elementary undergraduate
   4. Elementary graduate
   5. High school undergraduate
   6. High school graduate
   7. Post- secondary
   8. College undergraduate
   9. College graduate
   10. Post-graduate degree completed
5. Occupation: Which of the following best describes your main work status over the past 12 months?
   1. Government employee
   2. Non-government employee
   3. Self-employed
   4. Student
   5. Housekeeper
   6. Retired
   7. Unemployed, able to work
   8. Unemployed, unable to work
6. Monthly income. Can you let me know which category your monthly income falls under?
   1. No income
   2. Below 3,499

c. 3,500 to 4,999

d. 5,000 to 8,499

e. 8,500 to 19,999

f. 20,000 to 20,999

g. 21,000 to 29,999

h. 30,000 to 39,999

i. 40,000 - 49,999

j. 50,000 or higher

# *page 3*

**SECTION B: Smoking and quitting smoking**

# You will now be asked about smoking.

1. Which of these best describes you?
   1. I smoke everyday. ******If this is the answer, proceed to the Question#8 and Question#9 of Section B**
   2. I smoke at least once a month, but not every day. ******If this is the answer, proceed to the Question#10 of Section B**
   3. I used to smoke but I don’t smoke now; ******If this is the answer, proceed to the Question#11 of Section B**
   4. I have never smoked. ******If this is the answer, proceed to the Question#11 of Section B**
2. **If you are a smoker, Ask if Q7=a**
   1. When did you start smoking?
      1. Year
   2. Do you buy cigarettes...
      1. by the stick?
      2. by the pack?
   3. Do you also use e-cigarettes? (*dual use)*
      1. *No*
      2. *Yes*
   4. If yes, how often do you use e-cigarettes? Ask if Q8c=ii.Yes
3. 1-2 times a day
4. 3-4 times a day
5. 5 or more times a day
   1. If yes, what do you use on your ENDS device (electronic nicotine delivery system)? Ask if Q8c=ii.Yes
6. E-liquids
7. free base nicotine
8. Others *will specify*
9. If you’re a smoker, which of the following best describes your thinking about quitting smoking? **Ask if Q7=a**
10. I have thought about quitting but not seriously and haven’t cut down or tried to
11. I have thought seriously about wanting to quit in the next six months but I haven’t done anything yet
12. I intend to quit in the next six months and taking the steps to do so)
13. I am currently in the process of quitting/cutting down
14. I have tried quitting but keep starting again
15. **If you’re an occasional smoker, Ask if Q7=b**
    1. When did you start smoking?
       1. Year:
    2. Do you buy cigarettes…
       1. by the stick?
       2. by the pack?
    3. Do you also use e-cigarettes? (*dual use)*
       1. *No*
       2. *Yes*
    4. If yes, how often do you use e-cigarettes? Ask if Q10c=ii.Yes
       1. 1-2 times a day
       2. 3-4 times a day
       3. 5 or more times a day
    5. If yes, what do you use on your ENDS device (electronic nicotine delivery system)? Ask if Q10c=ii.Yes
       1. E-liquids
       2. free base nicotine
       3. Others *will specify*
    6. Which of the following best describes your thinking about quitting smoking?
16. I have thought about quitting but not seriously and haven’t cut down or tried to
17. I have thought seriously about wanting to quit in the next six months but I haven’t done anything yet
18. I intend to quit in the next six months and taking the steps to do so)
19. I am currently in the process of quitting/cutting down
20. I have tried quitting but keep starting again

# If you’re a non-smoker, which one best describes you? Ask if Q7=c,d

- 1. Non-smoker who recently quit (I have recently quit, less than 12 months ago)
  2. Non-smoker who recently quit cigarettes with ENDS (e-cigarette, vape) (I recently quit smoking with and are currently using an e-cigarette/vape)
     1. Which brand do you use? Ask if Q11=b
        1. E-liquids
        2. free base nicotine
        3. Others *will specify*
     2. How often do you use e-cigarettes? Ask if Q11=b
        1. 1-2 times a day
        2. 3-4 times a day
        3. 5 or more times a day
  3. Non-smoker who quit in the last ten years (I have quit smoking for good)
  4. Never smoker (I have never smoked a cigarette)
  5. Non-smoker, vaper (I don’t smoke and have never smoked a cigarette, I only use an e-cigarette/vape)
     1. Which brand do you use? Ask if Q11=e
        1. E-liquids
        2. free base nicotine
        3. Others *will specify*
     2. How often do you use e-cigarettes? Ask if Q11=e
        1. 1-2 times a day
        2. 3-4 times a day
        3. 5 or more times a day

# *page 4*

**SECTION C:**

In the next few questions, I will show you a few cigarette packs. Please take a few minutes to look at the packs. The exercise will be asked to compare the different packs shown on different measures. **Select the pack that corresponds to your answer in the dropdown menu.**

| ****Kindly make sure that packs are shown at all times while they are answering the question below.**** | **Pack 1** | **Pack 2** | **Pack 3** | **Pack 4** | **Pack 5** | **Pack 6** |
| --- | --- | --- | --- | --- | --- | --- |
| **1. Looking at these six cigarette packs, please indicate which pack you think is the... Most appealing overall and the Least appealing overall?** |  |  |  |  |  |  |
| Most visually appealing overall |  |  |  |  |  |  |
| Least visually appealing overall |  |  |  |  |  |  |
| **2. Looking at these six cigarette packs, we’d now like you to indicate which pack looks like it contains the... Highest quality cigarettes and the Lowest quality cigarettes?** | **Pack 1** | **Pack 2** | **Pack 3** | **Pack 4** | **Pack 5** | **Pack 6** |
| Highest quality cigarettes |  |  |  |  |  |  |
| Lowest quality cigarettes |  |  |  |  |  |  |
| **3. Looking at these six cigarette packs, we’d now like you to indicate which pack looks like it contains cigarettes that are the... Most harmful and the Least harmful to health?** | **Pack 1** | **Pack 2** | **Pack 3** | **Pack 4** | **Pack 5** | **Pack 6** |
| Most harmful to health |  |  |  |  |  |  |
| Least harmful to health |  |  |  |  |  |  |
| **4. Looking at these six**  **cigarette packs, we’d**  **now like you to indicate which pack looks like it would contain cigarettes that would be... Easiest to quit and Hardest to quit** | **Pack 1** | **Pack 2** | **Pack 3** | **Pack 4** | **Pack 5** | **Pack 6** |
| Easiest to quit |  |  |  |  |  |  |
| Hardest to quit |  |  |  |  |  |  |
| **5. Looking at these six cigarette packs, we’d now like you to indicate which pack looks like it contains cigarettes which you would be more likely to smoke?** | **Pack 1** | **Pack 2** | **Pack 3** | **Pack 4** | **Pack 5** | **Pack 6** |
| I would not smoke this |  |  |  |  |  |  |
| I would smoke this |  |  |  |  |  |  |
| **6. Looking at these six cigarette packs, we’d now like you to indicate which pack looks more effective in discouraging non-users from initiating tobacco use?** | **Pack 1** | **Pack 2** | **Pack 3** | **Pack 4** | **Pack 5** | **Pack 6** |
| Most effective |  |  |  |  |  |  |
| Least effective |  |  |  |  |  |  |

# *page 5*

****Kindly make sure that packs are shown at all times while they are answering the question below.****

**SECTION D: In the next few questions, I will show you a few cigarette packs. Please look at the specified pack and answer the questions about the pack and the plain packaging warning labels on the specified cigarette pack. Each pack has a different design and plain packaging warning labels that we would like you to look at. Select the appropriate box of your response or to what extent you agree.**

| **Pack 1**  **Looking on this first cigarette pack,** | **Strongly Agree** | **Agree** | **Neutral** | **Disagree** | **Strongly Disagree** |
| --- | --- | --- | --- | --- | --- |
| 1. Overall, to what extent do you agree that this pack design is appealing to you (i.e. you like the pack/ pack is attractive/)? |  |  |  |  |  |
| 2. Overall, to what extent do you agree that this pack design encourages you to try smoking or buy the pack? |  |  |  |  |  |
| 3. Overall, to what extent do you agree that you would like to try smoking the cigarettes contained in this pack? |  |  |  |  |  |
| 4. Overall, to what extent do you agree that you would like to be seen with this pack? |  |  |  |  |  |
| 5. Overall, to what extent do you agree that smoking the cigarettes in this pack is harmful to your health? |  |  |  |  |  |
| 6. Overall, to what extent do you agree that the health warnings labels on the front of each of these packs are noticeable? |  |  |  |  |  |
| 7. Overall, to what extent  do you agree that the health warnings labels on the front of each of these packs stands out to you/ catches your attention? |  |  |  |  |  |
| 8. Overall, to what extent do you agree that each of these packs make you ‘stop and think about the harmful effects of smoking’ when you look at them? |  |  |  |  |  |
| 9. Overall, to what extent do you agree that the message of the health warnings label on this pack is easy to understand? |  |  |  |  |  |

# *page 6*

**SECTION D: In the next few questions, I will show you a few cigarette packs. Please look at the specified pack and answer the questions about the pack and the plain packaging warning labels on the specified cigarette pack. Each pack has a different design and plain packaging warning labels that we would like you to look at. Select the appropriate box of your response or to what extent you agree.**

| **Pack 2**  **Looking on this second cigarette pack,** | **Strongly Agree** | **Agree** | **Neutral** | **Disagree** | **Strongly Disagree** |
| --- | --- | --- | --- | --- | --- |
| 10. Overall, to what extent do you agree that this pack design is appealing to you (i.e. you like the pack/ pack is attractive/)? |  |  |  |  |  |
| 11. Overall, to what extent do you agree that this pack design encourages you to try smoking or buy the pack? |  |  |  |  |  |
| 12. Overall, to what extent do you agree that you would like to try smoking the cigarettes contained in this pack? |  |  |  |  |  |
| 13. Overall, to what extent do you agree that you would like to be seen with this pack? |  |  |  |  |  |
| 14. Overall, to what extent do you agree that smoking the cigarettes in this pack is harmful to your health? |  |  |  |  |  |
| 15. Overall, to what extent do you agree that the health warnings labels on the front of each of these packs are noticeable? |  |  |  |  |  |
| 16. Overall, to what extent do you agree that the health warnings labels on the front of each of  these packs stands out to you/ catches your attention? |  |  |  |  |  |
| 17. Overall, to what extent do you agree that each of these packs make you ‘stop and think about the harmful effects of smoking’ when you look at them? |  |  |  |  |  |
| 18. Overall, to what extent do you agree that the message of the health warnings label on this pack is easy to understand? |  |  |  |  |  |

# *page 7*

**SECTION D: In the next few questions, I will show you a few cigarette packs. Please look at the specified pack and answer the questions about the pack and the plain packaging warning labels on the specified cigarette pack. Each pack has a different design and plain packaging warning labels that we would like you to look at. Select the appropriate box of your response or to what extent you agree.**

| **Pack 3**  **Looking on this third cigarette pack,** | **Strongly Agree** | **Agree** | **Neutral** | **Disagree** | **Strongly Disagree** |
| --- | --- | --- | --- | --- | --- |
| 19. Overall, to what extent do you agree that this pack design is appealing to you (i.e. you like the pack/ pack is attractive/)? |  |  |  |  |  |
| 20. Overall, to what extent do you agree that this pack design encourages you to try smoking or buy the pack? |  |  |  |  |  |
| 21. Overall, to what extent do you agree that you would like to try smoking the cigarettes contained in this pack? |  |  |  |  |  |
| 22. Overall, to what extent  do you agree that you would like to be seen with this pack? |  |  |  |  |  |
| 23. Overall, to what extent do you agree that smoking the cigarettes in this pack is harmful to your health? |  |  |  |  |  |
| 24. Overall, to what extent do you agree that the health warnings labels on the front of each of these packs are noticeable? |  |  |  |  |  |
| 25. Overall, to what extent do you agree that the health warnings labels on the front of each of these packs stands out to you/ catches your attention? |  |  |  |  |  |
| 26. Overall, to what extent do you agree that each of these packs make you ‘stop and think about the harmful effects of smoking’ when you look at them? |  |  |  |  |  |
| 27. Overall, to what extent do you agree that the message of the health warnings label on this pack is easy to understand? |  |  |  |  |  |

# *page 8*

**SECTION D: In the next few questions, I will show you a few cigarette packs. Please look at the specified pack and answer the questions about the pack and the plain packaging warning labels on the specified cigarette pack. Each pack has a different design and plain packaging warning labels that we would like you to look at. Select the appropriate box of your response or to what extent you agree.**

| **Pack 4**  **Looking on this fourth cigarette pack,** | **Strongly Agree** | **Agree** | **Neutral** | **Disagree** | **Strongly Disagree** |
| --- | --- | --- | --- | --- | --- |
| 28. Overall, to what extent do you agree that this pack design is appealing to you (i.e. you like the pack/ pack is attractive/)? |  |  |  |  |  |
| 29. Overall, to what extent do you agree that this pack design encourages you to try smoking or buy the pack? |  |  |  |  |  |
| 30. Overall, to what extent do you agree that you would like to try smoking the cigarettes contained in this pack? |  |  |  |  |  |
| 31. Overall, to what extent do you agree that you would like to be seen with this pack? |  |  |  |  |  |
| 32. Overall, to what extent do you agree that smoking the cigarettes in this pack is harmful to your health? |  |  |  |  |  |
| 33. Overall, to what extent do you agree that the health warnings labels on the front of each of these packs are noticeable? |  |  |  |  |  |
| 34. Overall, to what extent do you agree that the health warnings labels on the front of each of  these packs stands out  to you/ catches your attention? |  |  |  |  |  |
| 35. Overall, to what extent do you agree that each of these packs make you ‘stop and think about the harmful effects of smoking’ when you look at them? |  |  |  |  |  |
| 36. Overall, to what extent do you agree that the message of the health warnings label on this pack is easy to understand? |  |  |  |  |  |

# *page 9*

**SECTION D: In the next few questions, I will show you a few cigarette packs. Please look at the specified pack and answer the questions about the pack and the plain packaging warning labels on the specified cigarette pack. Each pack has a different design and plain packaging warning labels that we would like you to look at. Select the appropriate box of your response or to what extent you agree.**

| **Pack 5**  **Looking on this fifth cigarette pack,** | **Strongly Agree** | **Agree** | **Neutral** | **Disagree** | **Strongly Disagree** |
| --- | --- | --- | --- | --- | --- |
| 37. Overall, to what extent do you agree that this pack design is appealing to you (i.e. you like the pack/ pack is attractive/)? |  |  |  |  |  |
| 38. Overall, to what extent do you agree that this pack design encourages you to try smoking or buy the pack? |  |  |  |  |  |
| 39. Overall, to what extent do you agree that you would like to try smoking the cigarettes contained in this pack? |  |  |  |  |  |
| 40. Overall, to what extent do you agree that you would like to be seen with this pack? |  |  |  |  |  |
| 41. Overall, to what extent do you agree that smoking the cigarettes in this pack is harmful to your health? |  |  |  |  |  |
| 42. Overall, to what extent do you agree that the health warnings labels on the front of each of these packs are noticeable? |  |  |  |  |  |
| 43. Overall, to what extent do you agree that the health warnings labels on the front of each of  these packs stands out  to you/ catches your attention? |  |  |  |  |  |
| 44. Overall, to what extent do you agree that each of these packs make you ‘stop and think about the harmful effects of smoking’ when you look at them? |  |  |  |  |  |
| 45. Overall, to what extent do you agree that the message of the health warnings label on this pack is easy to understand? |  |  |  |  |  |

# *page 10*

**SECTION D: In the next few questions, I will show you a few cigarette packs. Please look at the specified pack and answer the questions about the pack and the plain packaging warning labels on the specified cigarette pack. Each pack has a different design and plain packaging warning labels that we would like you to look at. Select the appropriate box of your response or to what extent you agree.**

| **Pack 6**  **Looking on this sixth cigarette pack,** | **Strongly**  **Agree** | **Agree** | **Neutral** | **Disagree** | **Strongly Disagree** |
| --- | --- | --- | --- | --- | --- |
| 46. Overall, to what extent do you agree that this pack design is appealing to you (i.e. you like the pack/ pack is attractive/)? |  |  |  |  |  |
| 47. Overall, to what extent do you agree that this pack design encourages you to try smoking or buy the pack? |  |  |  |  |  |
| 48. Overall, to what extent do you agree that you would like to try smoking the cigarettes contained in this pack? |  |  |  |  |  |
| 49. Overall, to what extent do you agree that you would like to be seen with this pack? |  |  |  |  |  |
| 50. Overall, to what extent do you agree that smoking the cigarettes in this pack is harmful to your health? |  |  |  |  |  |
| 51. Overall, to what extent do you agree that the health warnings labels on the front of each of these packs are noticeable? |  |  |  |  |  |
| 52. Overall, to what extent do you agree that the health warnings labels on the front of each of these packs stands out to you/ catches your attention? |  |  |  |  |  |
| 53. Overall, to what extent do you agree that each of these packs make you ‘stop and think about the harmful effects of smoking’ when you look at them? |  |  |  |  |  |
| 54. Overall, to what extent do you agree that the message of the health warnings label on this pack is easy to understand? |  |  |  |  |  |

# *page 11*

**55. Thinking about the message on the health warning label that was on the pack, what do you recall seeing? List the one key message that you can think of?**

# *page 12*

**SECTION E: In the next few questions, we would like to know what you think about a scenario where plain or standardized packaging is introduced in the Philippines.**

| **Perception of Filipinos towards introducing standardized plain tobacco packaging** | **Strongly Agree** | **Agree** | **Neutral** | **Disagree** | **Strongly Disagree** |
| --- | --- | --- | --- | --- | --- |
| 1. Standardized plain packaging requires all tobacco products to be placed in unattractive packaging, without any promotional information (e.g. logos, colors and imagery) and carries health warnings.”  *To what extent do you agree that tobacco products (e.g. cigarettes, cigars, ang roll-your-own tobacco, e- cigarettes, vape products etc.) should come in standardized plain packaging?* |  |  |  |  |  |
| 2. Tobacco companies often use tobacco packaging for promotional purposes. Because of this, some health authorities in other countries have proposed that tobacco should be sold in plain packaging. This means that all packs would look the same with logos and colors removed. The brand name in plain text and pictorial health warnings would still remain on the packs.  *Overall, to what extent do you agree or disagree with this standardized plain packaging proposal?* |  |  |  |  |  |
| 3. To what extent do you agree that current health warning labels on tobacco packaging (e.g. cigarettes, cigars, etc.) of the Philippines should be larger than what it currently is? |  |  |  |  |  |
| 4. To what extent do you agree that plain packaging of tobacco products can decrease tobacco use? |  |  |  |  |  |
| 5. To what extent do you agree that plain packaging is relevant to the Philippine context? |  |  |  |  |  |
| 6. To what extent do you agree it is possible to adopt plain packaging in the Philippines? |  |  |  |  |  |

# *page 13*

**SECTION F.**

We will give possible reasons for introducing plain packaging in the Philippines. Select your best applicable response.

| **Do you think introducing standardized plain packaging in tobacco cigarette products will:** | **Strongly Agree** | **Agree** | **Neutral** | **Disagree** | **Strongly Disagree** |
| --- | --- | --- | --- | --- | --- |
| 56. Reduce the attractiveness and appeal |  |  |  |  |  |
| 57. Prevent advertisement and promotion on tobacco products packages |  |  |  |  |  |
| 58. Reduce ability of tobacco products packages to mislead consumers (with brand variants such as mild, smooth, lights) |  |  |  |  |  |
| 59. Increase the noticeability and effectiveness of the pictorial health warnings |  |  |  |  |  |
| 60. Increase recall of the pictorial health warnings |  |  |  |  |  |
| 61. Affect consumer perceptions of the attractiveness of the tobacco products |  |  |  |  |  |
| 62. Affect consumer perceptions of the relative safety of the tobacco products |  |  |  |  |  |
| 63. Reduce youth experimentation with use of tobacco products |  |  |  |  |  |
| 64. Prevent the use of brand variants (lights, mild, smooth) of tobacco products as a promotional tool |  |  |  |  |  |
| 65. Prevent branding and glamorization especially targeted towards youth (specific colors, design and descriptors like jazz, cool) |  |  |  |  |  |
| 66. Promote quitting among current users |  |  |  |  |  |
| 67. More clearly inform consumers about the harmful effects of tobacco use |  |  |  |  |  |

# *page14*

**Thank you very much for participating in our survey. We appreciate your support to help improve tobacco control policies in the Philippines.**

Thinking of quitting? For questions about your own smoking habit, you could talk to your doctor or reach out to the Department of Health’s quitline: 165-364 or SMS 09212039534 or 09776277539.

# THIS IS THE END OF THE SURVEY.

# Supplementary File 2. Proposed Survey Layouts

**
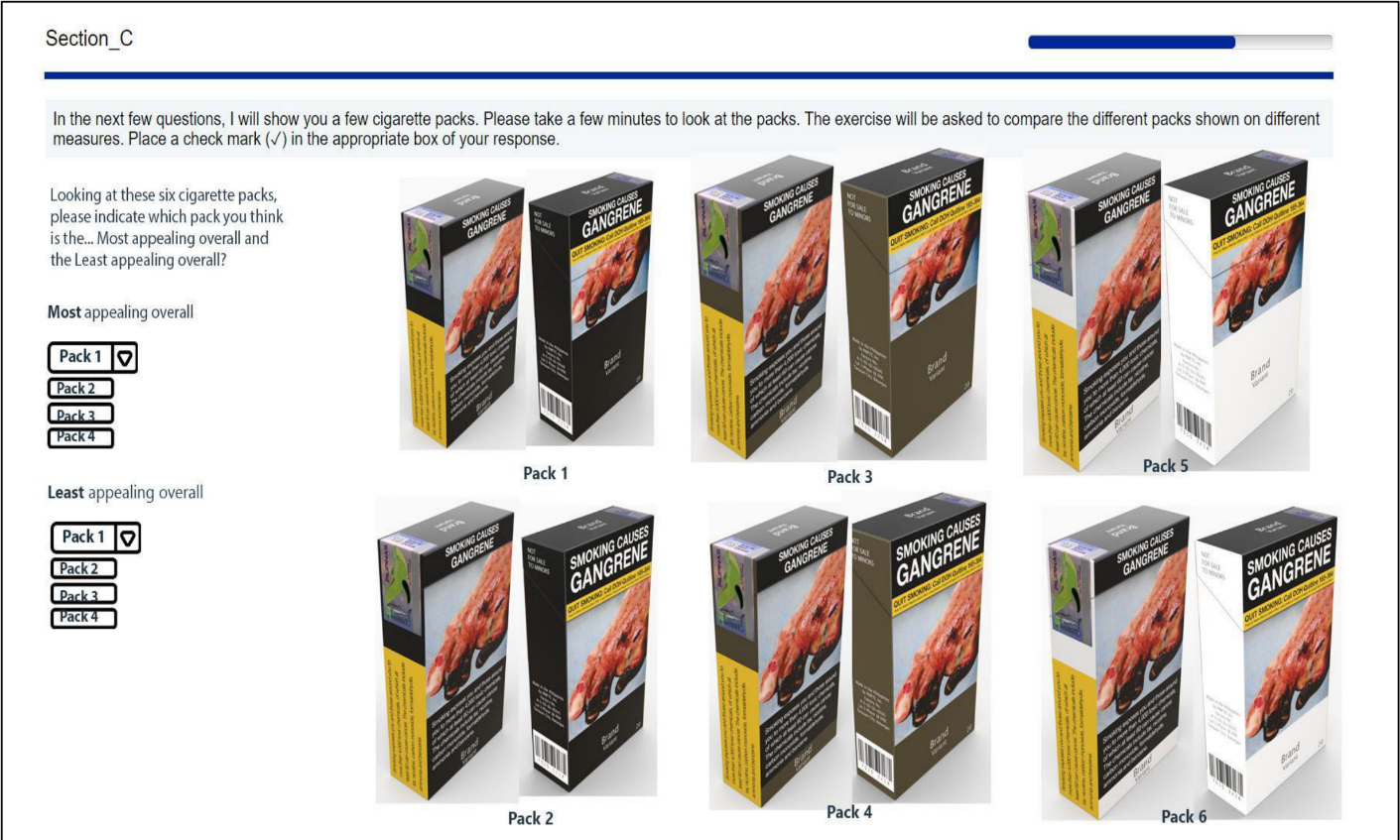
**

**Supplementary Figure 1. Proposed Survey Layout Sample 1**

**
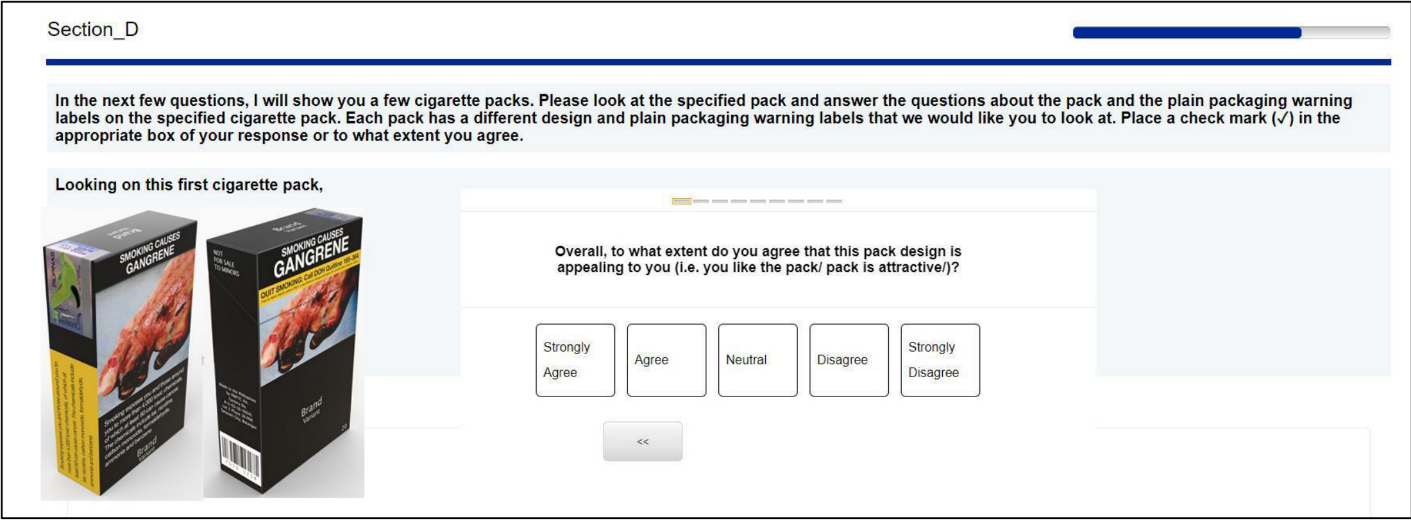
**

**Supplementary Figure 2. Proposed Survey Layout Sample 2**

**Supplementary File 3. Informed Consent Form - English and Filipino**

**INFORMED CONSENT FORM**ONLINE SURVEY VERSION

(ENGLISH VERSION)

Title of the Research Study: **Promoting the Tobacco Plain Packaging Bill: A study on local perception and readiness of Filipinos**

Name of Sponsor / Funder / Grant-Provider *[if applicable]*: The International Union Against Tuberculosis and Lung Disease (The Union)

Name of Principal Investigator (PI):  Ivyrose S. Baysic

Institution: Ateneo Policy Center (APC), Ateneo School of Government (ASoG), Ateneo de Manila University (ADMU)

Contact Information of PI: policycenter.asog@ateneo.edu

You are being asked to take part in a research study. Before you decide to participate or not, please read the informed consent form. The informed consent form indicates the purpose of the study, the possible risks and benefits of being in the study, and what you will have to do if you decide to participate.

If you do not understand what you are reading, do not agree to participate in the study. You may ask the principal investigator, whose contact details are listed above, to explain anything you do not understand, including any language contained in this form, or if you have any questions or concerns.

**What is the purpose of the study?**

The purpose of the study is to build and generate local evidence on introducing the standardized plain packaging of tobacco products and cigarette packs in the Philippines. Specifically, the study aims to 1) determine the perception of  Filipinos towards plain tobacco packaging in comparison to fully branded packaging in terms of health warnings (increased recall), product harm and strength, and smoking-related behavior (attitude, intention or initiation, and beliefs); 2) assess potential impact of plain packaging to smokers' attempt to quit, ease of quitting, and other quitting related cognitions; and 3) measure the reducing false and misleading information on plain packaging.

**Why am I being asked to participate in the study?**

You are being asked to join this study because it targets Filipino adults (both smokers and non-smokers) ages 18 to 65 years old residing in the Philippines.

**What will I be asked to do?**

You will be asked to complete an online survey. We will ask you questions that will provide us insights regarding the perceptions of Filipino adults on the standardized plain packaging on cigarette packs in the Philippines.

**How long will I be in the study?**

The online survey will take about 10-20 minutes to complete.

**Where will the study take place?**

You are free to answer the online survey at any place and time provided that you have a gadget or device with internet connection (i.e. computer or smartphone) on the Rakuten Insight platform.

**Are there any risks and what are they?**

There are little to no risks to your health, privacy, or safety posed by your participation in this study. You may experience  minor psychological or emotional discomfort brought by the images of graphic health warnings **.**

The study has low privacy risk, and does not entail gathering sensitive information. Your identity will not be released by Rakuten Insight to the ASoG  in any form without prior written consent.

If you are thinking of quitting or have questions about your own smoking habits, you may talk to your doctor or reach out to the Department of Health’s quit line: 165-364 or SMS 09212039534 or 09776277539.

If you may need psychological counseling, we can refer you, with your consent, to the Ateneo Bulatao Center ([bulataocenter.ls@ateneo.edu](mailto:bulataocenter.ls@ateneo.edu)), UGAT Foundation (<https://www.facebook.com/ugatfoundationinc/>), UGAT SandaLine (<https://www.facebook.com/UGATSandaLinePage/>), or the Philippine Mental Health Association. You may also refer to the following contact details of various organizations that provide mental health care services through this link: <https://bit.ly/2TZ8cDc>.

Kindly remember that you can opt to discontinue at any point or not answer a question if you feel uncomfortable.

**What are the benefits of participating in the study?**

Your participation in this study could help us identify and assess the perception of Filipinos towards standardized plain packaging. This will help inform policy recommendations that will help curb the smoking prevalence in the Philippines and eventually promote a healthier and a more productive society.

**What happens if I do not choose to join the research study?** **Can I stop or withdraw from the study even after it has started?**

You may choose to join the study or you may choose not to join the study. Your participation is voluntary. There is no penalty if you choose not to join the research study. You will not lose any benefits or advantages that you are now receiving or will receive in the future.

You can stop your participation in the research study and withdraw your data at any time even after it has started. There is no penalty or loss of benefits if you decide to do so.

If you no longer wish to be part of the research study, or if you want to withdraw your data so that the research will no longer use it, please contact the project leader using the contact details provided above indicating your request to withdraw and we will promptly delete all information that you have provided

**How will confidentiality be maintained and my privacy protected?** **What** **personal or identifiable data will you obtain? Who will have access to or see my data?**

The research team will comply with all pertinent privacy guidelines of the ADMU (implementing institution) and the Data Privacy Act of 2012. Any personal information will not be shared by Rakuten Insight to the ADMU.  The information you provide is confidential. Your full name and other personal information will not appear in any report or publication of this research..

**Will I have to pay for anything?**

Participation in this study is free and will not entail any cost.

# Will I be paid for participating in this study?

There will be no compensation for participating in this study. Members of the Rakuten Insight who completed the survey will earn rewards points from Rakuten Insight. Only the Rakuten Insights determines the amount of points a Member can earn from each survey.

# Who can I call for questions about the study or if I’m concerned about my rights as a research participant?

If you have questions or concerns regarding the study and your participation in it, contact the Principal Investigator listed on page 1 of this form.

If a member of the research team cannot be reached or you want to talk to someone other than those working on the study, you may contact the University Research Ethics Office at the Ateneo de Manila University by calling (+63) 945 213 6758 for any question, concern, or complaint about your rights as a research subject.

**Certificate of Consent**

*[This section is mandatory and should be written in first person language. Fill in the blanks with the appropriate information.]*

I have been invited to participate in a study. I have read the information about the study, or it has been read to me. I have had the opportunity to ask questions about it and they have been answered to my satisfaction.

I consent voluntarily to be a participant in this study.

Signature of Participant:

Printed Name of Participant:

Date (month/day/year):

I have witnessed the accurate reading of the consent form to the potential participant, and the individual has had the opportunity to ask questions. I confirm that the individual has given consent freely.

Signature of Witness:

Printed Name of Witness:

Date (month/day/year):

**Statement by the researcher/person obtaining consent**

I confirm that the participant was given an opportunity to ask questions about the study, and all the questions asked by the participant have been answered correctly and to the best of my ability. I confirm that the individual has not been coerced into giving consent, and the consent has been given freely and voluntarily.

A copy of this document has been provided to the participant.

Signature of Researcher /person taking the consent:

Print Name of Researcher/person taking the consent:

Date (month/day/year):

**INFORMED CONSENT FORM
ONLINE SURVEY VERSION**

**(FILIPINO VERSION)**

Pangalan ng Pag-aaral: Promoting the Tobacco Plain Packaging Bill: A study on local perception and readiness of Filipinos

Pangalan ng Sponsor: The International Union Against Tuberculosis and Lung Disease (The Union)

Pangalan ng Principal Investigator (PI): Ivyrose S. Baysic

Institusyon: Ateneo Policy Center (APC), Ateneo School of Government (ASoG), Ateneo de Manila University (ADMU)

Email Address: policycenter.asog@ateneo.edu

______________________________________________________________________

Iniimbitahan kang maging bahagi ng pag-aaral na ito, pero bago ang lahat, siguruhing nabasa ang laman ng *informed  consent form.* Nakapaloob sa *informed consent form* ang layunin ng pag-aaral, ang mga posibleng panganib at benepisyo  ng pagiging bahagi ng pananaliksik, at kung ano ang mga kailangan gawin bilang kalahok.

Huwag ituloy ang pagsali kung may hindi naiintindihan sa anumang bahagi ng pag-aaral. Pwedeng magtanong para maipaliwanag sa iyo ang anumang hindi mo naiintindihan, kabilang na ang wika o lenggwaheng nakapaloob sa form na ito, o kung may mga tanong ka tungkol sa mismong pag-aaral.

**Ano ang layunin ng pag-aaral?**

Layunin ng pag-aaral na kumuha at bumuo ng mga ebidensya para sa pagpapakilala ng standardized plain packaging sa mga pakete ng sigarilyo at tobacco sa Pilipinas. Layunin din nito na 1) malaman ang opinyon o perspektibo ng mga Pilipino pagdating sa standardized plain packaging sa Pilipinas sa aspeto ng health warnings, panganib ng produkto, at iba’t ibang ugali, intensyon, paniniwala pagdating sa paninigarilyo; 2) ano ang pwedeng epekto ng plain packaging sa mga pananaw ng smokers pagdating sa kanyang desisyon na tumigil sa paninigarilyo, gaano kadali sa kanila na tumigil sa paninigarilyo, at iba pang aspeto ng pagtigil sa paninigarilyo; at 3) suriin kung paano nakakatulong ang standardized plain packaging para mabawasan ang mga maling impormasyon at detalye pagdating sa isang sigarilyo.

**Bakit ako iniimbitahang maging bahagi ng pag-aaral na ito?**

Sinasama ka sa pag-aaral na ito dahil pasok ka sa target ng pag-aaral na mga Pilipinong edad 18 hanggang 65 taong  gulang, naninigarilyo man o hindi.

**Ano ang mga kailangan kong gawin?**

Kailangan mong sumagot ng isang survey. Layunin ng mga tanong na makuha ang pananaw at pang-unawa mo sa standardized plain packaging na nasa pakete o kaha ng mga sigarilyo sa Pilipinas.

**Gaano katagal ako kailangan para sa pag-aaral?**

Nasa 10 hanggang 30 minuto ang kailangan para masagutan nang buo ang mga tanong sa survey.

**Saan gaganapin ang pag-aaral?**

Ang survey ay maaaring sagutin sa Rakuten Insight platform, sa iyong bahay na ikaw ay may access sa device na may internet.

**May mga panganib ba at ano ang mga ito?**

Halos walang panganib na dala sa iyong kalusugan, privacy, at kaligtasan ang pagiging kalahok sa pag-aaral na ito. Maaaring  magdulot ng kaunting psychological o emotional discomfort ang mga halimbawang graphic health warnings na kasama sa  survey na ito.

Ang pag aaral ay may mababang privacy risk. Hindi namin kukunin ang iyong sensitibong impormasyon. Hindi ibibigay ng Rakuten Insight sa ASoG at ano mang iyong pagkakakilanlan.

Kung naiisip mong tumigil o may mga tanong ka tungkol sa kung paano ka puwedeng tumigil sa iyong paninigarilyo,  puwedeng kumonsulta sa doktor o tumawag sa quit line ng Department of Health (DOH): 165-364 o SMS 09212039534 o  09776277539.

Kung kailangan ng psychological counseling, maaari namin kayong ilapit sa Ateneo Bulatao Center  (bulataocenter.ls@ateneo.edu), UGAT Foundation (https://www.facebook.com/ugatfoundationinc/), UGAT SandaLine  (https://www.facebook.com/UGATSandaLinePage/), o sa Philippine Mental Health Association. Maaari rin ninyong i-check  ang mga sumusunod na organisasyong nagbibigay ng mental health care services: https://bit.ly/2TZ8cDc.

Anumang oras ay puwede kang tumigil sa pagsagot kung hindi ka na komportable sa mga tanong.

**Ano ang mga benepisyo ng pagiging kalahok sa pag-aaral?**

Makakatulong ka sa pagtukoy ng mga perspektibo nga mga Pilipino pagdating sa standardized plain packaging ng mga pakete ng sigarilyo.  Makakatulong ka rin sa pagbuo ng mga polisiya at rekomendasyon para mapigilan ang pagdami ng mga naninigarilyo sa  bansa at para masigurong malusog at produktibo ang lipunan natin.

**Anong mangyayari kung piliin kong hindi maging bahagi ng pag-aaral? Maaari ba akong tumigil o mag withdraw mula sa pag-aaral kahit na nakapagsimula na ako?**

Nasa sa iyo kung sasali ka o hindi sa pag-aaral. Boluntaryo dapat ang partisipasyon mo dito. Walang parusa kung piliin man  ninyong hindi maging bahagi ng pag-aaral na ito. Wala ring mawawala sa iyo anuman ang maging desisyon mo sa  pakikilahok.

Anumang oras, pwede mong ihinto ang iyong partisipasyon sa pag-aaral at kunin ang ibinigay mong impormasyon. Walang  parusa kung gagawin mo ito.

Kung hindi mo na gustong maging bahagi ng pag-aaral na ito, o kung nais mong kunin ang impormasyon na binigay mo  para hindi ito magamit sa pag-aaral, puwede kang makipag-ugnayan sa project leader gamit ang mga contact details na  nasa itaas. Buburahin agad ng mga mananaliksik ang lahat ng binigay mong impormasyon.

**Paano masisiguro na mananatiling kumpidensiyal at pribado ang aking impormasyon? Anong mga personal na  datos ang inyong kunin? Sino ang may access o makakakita sa mga ito?**

Sumusunod ang mga mananaliksik sa mga ipinapatupad na privacy guidelines ng Ateneo de Manila University (implementing  institution) at ng Data Privacy Act. Anumang personal na impormasyong ibibigay mo ay hindi ibabahagi ng Rakuten Insight sa Ateneo de Manila University. Mananatiling confidential ang impormasyong ibibigay mo. Hindi lalabas sa anumang report o publikasyon ang buong  pangalan mo at iba pang personal na impormasyon na binigay mo.

**Kailangan ko bang magbayad para dito?**

Libre ang partisipasyon sa pag-aaral na ito. Walang kailangang bayaran.

**Babayaran ba ako sa partisipasyon ko sa pag-aaral?**

Walang compensation sa paglahok sa pag-aaral na ito. Ang mga miyembro ng Rakuten Insight na sumagot sa survey at makakakuha ng rewards points mula sa Rakuten Insight. Ang Rakuten Insight lang ang makakapabigay ng detalye kung ilan ang rewards points na makukuha ng isang member sa bawat isang survey na matatapos niya.

**Sino ang puwede kong tawagan kung may tanong ako tungkol sa mismong pag-aaral o sa aking mga karapatan  bilang kalahok ng pananaliksik na ito?**

Kung may iba pang tanong tungkol sa pag-aaral at ang kabuoang partisipasyon mo dito, puwede kang i-contact ang principal  investigator sa mga numerong makikita sa Page 1 ng form na ito.

Kung hindi sumasagot ang mga mananaliksik o kung nais mong makipag-usap sa kung sinoman labas sa grupo ng mga  mananaliksik, puwede mong tawagan ang University Research Ethics Office ng Ateneo de Manila University sa numerong  (+63) 945 213 6758 para sa mga katanungan, isyu o reklamo hinggil sa mga karapatan mo bilang kalahok ng pag-aaral na  ito.

**Sertipiko ng Pahintulot** (Certificate of Consent)

- Naimbitahan akong maging kalahaok (participant) ng isang pag-aaral tungkol sa standardized plain packaging na makikita  sa mga pakete o kaha ng sigarilyo. Sa pag-aaral na ito, hinihiling na sumagot ako ng isang survey.
- Nabasa ko nang buo ang lahat ng impormasyon ukol sa pag-aaral, o binasa ang nilalaman nito sa akin. Nabigyan ako  ng oportunidad na magtanong tungkol sa sakop ng pag-aaral at naging sapat ang paliwanag na ibinigay sa akin para  maintindihan ko ang mga ito.
- Ibinibigay ko ang aking pahintulot na maging kalahok sa pag-aaral na ito.

Pirma ng Kalahok:

Printed na Pangalan ng Kalahok:

Petsa (buwan/araw/taon):

**Pahayag ng mananaliksik/ taong kumukuha ng pahintulot**

- Kinukumpirma ko na ang kalahok (participant) ay nabigyan ng oportunidad na magtanong tungkol sa sakop ng pag aaral, at lahat ng mga tanong ng kalahok ay nasagot nang tama sa abot ng aking makakaya. Kinukumpirma ko na hindi  pinilit ang kalahok na ibigay ang kaniyang pahintulot. Malaya at boluntaryo niya itong ibinigay sa akin.  • Binigyan ang kalahok ng kopya ng dokumentong ito para sa kaniyang kaalaman.

Pirma ng Mananaliksik/ taong kumukuha ng pahintulot:

Printed na Pangalan ng Mananaliksik/ taong kumukuha ng pahintulot:

Petsa (buwan/araw/taon):

# Supplementary File 4. Checklist for Reporting of Survey Studies (CROSS) Checklist

| **Section/topic** | **Item** | **Item description** | **Reported on page #** |
| --- | --- | --- | --- |
| **Title and abstract** | | |  |
| Title and abstract | 1a | State the word “survey” along with a commonly used term in title or abstract to introduce the study’s design. | 1 |
|  | 1b | Provide an informative summary in the abstract, covering background, objectives, methods, findings/results, interpretation/discussion, and conclusions. | 2 |
| **Introduction** | | |  |
| Background | 2 | Provide a background about the rationale of study, what has been previously done, and why this survey is needed. | 1 - 2 |
| Purpose/aim | 3 | Identify specific purposes, aims, goals, or objectives of the study. | 2 |
| **Methods** | | |  |
| Study design | 4 | Specify the study design in the methods section with a commonly used term (e.g., cross-sectional or longitudinal). | 2 |
|  | 5a | Describe the questionnaire (e.g., number of sections, number of questions, number and names of instruments used). | 2 |
| Data collection methods | 5b | Describe all questionnaire instruments that were used in the survey to measure particular concepts. Report target population, reported validity and reliability information, scoring/classification procedure, and reference links (if any). | 2 - 3 |
|  | 5c | Provide information on pretesting of the questionnaire, if performed (in the article or in an online supplement). Report the method of pretesting, number of times questionnaire was pre-tested, number and demographics of participants used for pretesting, and the level of similarity of demographics between pre-testing participants and sample population. | 3 |
|  | 5d | Questionnaire if possible, should be fully provided (in the article, or as appendices or as an online supplement). | 2 |
| Sample characteristics | 6a | Describe the study population (i.e., background, locations, eligibility criteria for participant inclusion in survey, exclusion criteria). | 2 - 3 |
|  | 6b | Describe the sampling techniques used (e.g., single stage or multistage sampling, simple random sampling, stratified sampling, cluster sampling, convenience sampling). Specify the locations of sample participants whenever clustered sampling was applied. | 2 |
|  | 6c | Provide information on sample size, along with details of sample size calculation. | 2 |
|  | 6d | Describe how representative the sample is of the study population (or target population if possible), particularly for population-based surveys. | 2 |
| Survey  administration | 7a | Provide information on modes of questionnaire administration, including the type and number of contacts, the location where the survey was conducted (e.g., outpatient room or by use of online tools, such as SurveyMonkey). | 3 |
|  | 7b | Provide information of survey’s time frame, such as periods of recruitment, exposure, and follow-up days. | 3 - 4 |
|  | 7c | Provide information on the entry process:  –>For non-web-based surveys, provide approaches to minimize human error in data entry.  –>For web-based surveys, provide approaches to prevent “multiple participation” of participants. | 3 - 4 |
| Study preparation | 8 | Describe any preparation process before conducting the survey (e.g., interviewers’ training process, advertising the survey). | 3 - 4 |
| Ethical considerations | 9a | Provide information on ethical approval for the survey if obtained, including informed consent, institutional review board [IRB] approval, Helsinki declaration, and good clinical practice [GCP] declaration (as appropriate). | 3 - 4 |
|  | 9b | Provide information about survey anonymity and confidentiality and describe what mechanisms were used to protect unauthorized access. | 4 |
| Statistical  analysis | 10a | Describe statistical methods and analytical approach. Report the statistical software that was used for data analysis. | 4 |
|  | 10b | Report any modification of variables used in the analysis, along with reference (if available). | 4 |
|  | 10c | Report details about how missing data was handled. Include rate of missing items, missing data mechanism (i.e., missing completely at random [MCAR], missing at random [MAR] or missing not at random [MNAR]) and methods used to deal with missing data (e.g., multiple imputation). | None |
|  | 10d | State how non-response error was addressed. | None |
|  | 10e | For longitudinal surveys, state how loss to follow-up was addressed. | None |
|  | 10f | Indicate whether any methods such as weighting of items or propensity scores have been used to adjust for non-representativeness of the sample. | None |
|  | 10g | Describe any sensitivity analysis conducted. | None |
| **Results** | | |  |
| Respondent characteristics | 11a | Report numbers of individuals at each stage of the study. Consider using a flow diagram, if possible. | 3 - 4 |
|  | 11b | Provide reasons for non-participation at each stage, if possible. | None |
|  | 11c | Report response rate, present the definition of response rate or the formula used to calculate response rate. | None |
|  | 11d | Provide information to define how unique visitors are determined. Report number of unique visitors along with relevant proportions (e.g., view proportion, participation proportion, completion proportion). | 4 |
| Descriptive  results | 12 | Provide characteristics of study participants, as well as information on potential confounders and assessed outcomes. | 4 - 5 |
| Main findings | 13a | Give unadjusted estimates and, if applicable, confounder-adjusted estimates along with 95% confidence intervals and p-values. | 4 - 6 |
|  | 13b | For multivariable analysis, provide information on the model building process, model fit statistics, and model assumptions (as appropriate). | None |
|  | 13c | Provide details about any sensitivity analysis performed. If there are considerable amount of missing data, report sensitivity analyses comparing the results of complete cases with that of the imputed dataset (if possible). | None |
| **Discussion** | | |  |
| Limitations | 14 | Discuss the limitations of the study, considering sources of potential biases and imprecisions, such as non-representativeness of sample, study design, important uncontrolled confounders. | 7 |
| Interpretations | 15 | Give a cautious overall interpretation of results, based on potential biases and imprecisions and suggest areas for future research. | 8 |
| Generalizability | 16 | Discuss the external validity of the results. | 8 |
| **Other sections** | | |  |
| Role of funding source | 17 | State whether any funding organization has had any roles in the survey’s design, implementation, and analysis. | 14 |
| Conflict of interest | 18 | Declare any potential conflict of interest. | 14 |
| Acknowledgements | 19 | Provide names of organizations/persons that are acknowledged along with their contribution to the research. | 14 |

#

# Supplementary File 5. Socio-demographic profile of online survey respondents per smoking status

| **Supplementary Table 1. Socio-demographic profile of online survey respondents per smoking status** | | | | | | | | | |
| --- | --- | --- | --- | --- | --- | --- | --- | --- | --- |
| **Characteristics** | **Daily smokers** | | **Occasional Smokers** | | **Former Smokers** | | **Never Smokers or non-smokers** | | **Total** |
| **Gender** | **n** | **%** | **n** | **%** | **n** | **%** | **n** | **%** |  |
| Male | 355 | 69.7% | 198 | 52.5% | 231 | 43.0% | 201 | 34.8% | 985 |
| Female | 151 | 29.7% | 166 | 44.0% | 305 | 56.8% | 363 | 62.9% | 985 |
| Gender diverse | 3 | 0.6% | 13 | 3.4% | 1 | 0.2% | 13 | 2.3% | 30 |
| **Characteristics** | **Daily smokers** | | **Occasional Smokers** | | **Former Smokers** | | **Never Smokers or non-smokers** | | **Total** |
| **Age Group (years)** | **n** | **%** | **n** | **%** | **n** | **%** | **n** | **%** |  |
| 18-24 | 65 | 12.8% | 87 | 23.1% | 196 | 36.5% | 390 | 67.6% | 738 |
| 25-34 | 179 | 35.2% | 122 | 32.4% | 143 | 26.6% | 61 | 10.6% | 505 |
| 35-44 | 157 | 30.8% | 104 | 27.6% | 104 | 19.4% | 28 | 4.9% | 393 |
| 45-54 | 73 | 14.3% | 38 | 10.1% | 27 | 5.0% | 20 | 3.5% | 158 |
| 55-64 | 33 | 6.5% | 25 | 6.6% | 64 | 11.9% | 75 | 13.0% | 197 |
| 65-74 | 2 | 0.4% | 1 | 0.3% | 3 | 0.6% | 3 | 0.5% | 9 |
| **Characteristics** | **Daily smokers** | | **Occasional Smokers** | | **Former Smokers** | | **Never Smokers or non-smokers** | | **Total** |
| **Region** | **n** | **%** | **n** | **%** | **n** | **%** | **n** | **%** |  |
| NCR | 151 | 29.7% | 58 | 15.4% | 144 | 26.8% | 147 | 25.5% | 500 |
| CAR | 2 | 0.4% | 1 | 0.3% | 13 | 2.4% | 8 | 1.4% | 24 |
| I | 9 | 1.8% | 5 | 1.3% | 9 | 1.7% | 8 | 1.4% | 31 |
| II | 4 | 0.8% | 2 | 0.5% | 7 | 1.3% | 6 | 1.0% | 19 |
| III | 30 | 5.9% | 19 | 5.0% | 42 | 7.8% | 38 | 6.6% | 129 |
| IV-A | 59 | 11.6% | 29 | 7.7% | 91 | 16.9% | 80 | 13.9% | 259 |
| IV-B | 0 | 0.0% | 3 | 0.8% | 6 | 1.1% | 5 | 0.9% | 14 |
| V | 4 | 0.8% | 5 | 1.3% | 5 | 0.9% | 10 | 1.7% | 24 |
| VI | 33 | 6.5% | 28 | 7.4% | 35 | 6.5% | 25 | 4.3% | 121 |
| VII | 102 | 20.0% | 76 | 20.2% | 66 | 12.3% | 80 | 13.9% | 324 |
| VIII | 12 | 2.4% | 16 | 4.2% | 15 | 2.8% | 12 | 2.1% | 55 |
| IX | 9 | 1.8% | 13 | 3.4% | 15 | 2.8% | 31 | 5.4% | 68 |
| X | 36 | 7.1% | 45 | 11.9% | 26 | 4.8% | 29 | 5.0% | 136 |
| XI | 36 | 7.1% | 50 | 13.3% | 43 | 8.0% | 59 | 10.2% | 188 |
| XII | 17 | 3.3% | 18 | 4.8% | 12 | 2.2% | 18 | 3.1% | 65 |
| XIII | 5 | 1.0% | 9 | 2.4% | 6 | 1.1% | 19 | 3.3% | 39 |
| BARMM | 0 | 0.0% | 0 | 0.0% | 2 | 0.4% | 2 | 0.3% | 4 |
| **Characteristics** | **Daily smokers** | | **Occasional Smokers** | | **Former Smokers** | | **Never Smokers or non-smokers** | | **Total** |
| **Educational Attainment** | **n** | **%** | **n** | **%** | **n** | **%** | **n** | **%** |  |
| No grade completed | 0 | 0.0% | 0 | 0.0% | 1 | 0.2% | 2 | 0.3% | 3 |
| Elementary undergraduate | 1 | 0.2% | 2 | 0.5% | 1 | 0.2% | 1 | 0.2% | 5 |
| Elementary graduate | 2 | 0.4% | 0 | 0.0% | 0 | 0.0% | 1 | 0.2% | 3 |
| Highschool undergraduate | 7 | 1.4% | 5 | 1.3% | 5 | 0.9% | 18 | 3.1% | 35 |
| Highschool graduate | 60 | 11.8% | 24 | 6.4% | 68 | 12.7% | 91 | 15.8% | 243 |
| Post-secondary/Vocational | 5 | 1.0% | 6 | 1.6% | 11 | 2.0% | 21 | 3.6% | 43 |
| College undergraduate | 92 | 18.1% | 68 | 18.0% | 138 | 25.7% | 194 | 33.6% | 492 |
| College graduate | 324 | 63.7% | 255 | 67.6% | 302 | 56.2% | 235 | 40.7% | 1116 |
| Post-graduate | 18 | 3.5% | 17 | 4.5% | 11 | 2.0% | 14 | 2.4% | 60 |
| **Characteristics** | **Daily smokers** | | **Occasional Smokers** | | **Former Smokers** | | **Never Smokers or non-smokers** | | **Total** |
| **Occupation** | **n** | **%** | **n** | **%** | **n** | **%** | **n** | **%** |  |
| Government | 77 | 15.1% | 49 | 13.0% | 49 | 9.1% | 34 | 5.9% | 209 |
| Non-government/Private | 283 | 55.6% | 183 | 48.5% | 208 | 38.7% | 152 | 26.3% | 826 |
| Self-employed | 100 | 19.6% | 91 | 24.1% | 129 | 24.0% | 111 | 19.2% | 431 |
| Student | 10 | 2.0% | 24 | 6.4% | 72 | 13.4% | 169 | 29.3% | 275 |
| Housekeeper | 8 | 1.6% | 13 | 3.4% | 23 | 4.3% | 24 | 4.2% | 68 |
| Retired | 5 | 1.0% | 3 | 0.8% | 16 | 3.0% | 15 | 2.6% | 39 |
| Unemployed, able to work | 23 | 4.5% | 13 | 3.4% | 36 | 6.7% | 62 | 10.7% | 134 |
| Unemployed, unable to work | 3 | 0.6% | 1 | 0.3% | 4 | 0.7% | 10 | 1.7% | 18 |
| **Characteristics** | **Daily smokers** | | **Occasional Smokers** | | **Former Smokers** | | **Never Smokers or non-smokers** | | **Total** |
| **Monthly income (Php)** | **n** | **%** | **n** | **%** | **n** | **%** | **n** | **%** |  |
| No income | 12 | 2.4% | 10 | 2.7% | 45 | 8.4% | 131 | 22.7% | 198 |
| Below 3,499 | 11 | 2.2% | 9 | 2.4% | 22 | 4.1% | 70 | 12.1% | 112 |
| 3,500 to 4,999 | 16 | 3.1% | 9 | 2.4% | 29 | 5.4% | 34 | 5.9% | 88 |
| 5,000 to 8,499 | 34 | 6.7% | 16 | 4.2% | 54 | 10.1% | 60 | 10.4% | 164 |
| 8,500 to 19,999 | 100 | 19.6% | 88 | 23.3% | 131 | 24.4% | 118 | 20.5% | 437 |
| 20,000 to 20,999 | 52 | 10.2% | 60 | 15.9% | 59 | 11.0% | 50 | 8.7% | 221 |
| 21,000 to 29,999 | 73 | 14.3% | 53 | 14.1% | 78 | 14.5% | 43 | 7.5% | 247 |
| 30,000 to 39,999 | 75 | 14.7% | 59 | 15.6% | 52 | 9.7% | 25 | 4.3% | 211 |
| 40,000 - 49,999 | 66 | 13.0% | 39 | 10.3% | 36 | 6.7% | 19 | 3.3% | 160 |
| 50,000 or higher | 70 | 13.8% | 34 | 9.0% | 31 | 5.8% | 27 | 4.7% | 162 |
|  |  |  |  |  |  |  |  |  |  |
| Total | 509 | 100.0% | 377 | 100.0% | 537 | 100.0% | 577 | 100.0% | 2000 |
|  |  |  |  |  |  |  |  |  |  |

# Supplementary File 6. Smoking status of survey respondents

| **Supplementary Table 2. Smoking status of the survey respondents** | | |
| --- | --- | --- |
| **Smoking status** | **n** | **Percent** |
| I smoke everyday (Daily Smokers) | 509 | 25.5% |
| I smoke at least once a month (occasionally), but not every day (Occasional smokers) | 377 | 18.9% |
| I used to smoke but I don't smoke now (Former smokers) | 537 | 26.9% |
| I have never smoked (Never Smokers) | 577 | 28.9% |
| Total | 2,000 | 100.0% |

#

# Supplementary File 7. Smoking history of non-smoker respondents

| **Supplementary Table 3. Smoking history of non-smoker respondents** | | |
| --- | --- | --- |
| ***Characteristics*** | ***n*** | ***%*** |
| **If you’re a non-smoker, which one best describes you?** |  |  |
| Non-smokers who recently quit (I have recently quit, less than 12 months ago) | 180 | 16.2% |
| Non-smokers who recently quit cigarettes with ENDS (e-cigarette, vape) | 113 | 10.1% |
| Non-smokers who quit in the last ten years (I have quit smoking for good) | 207 | 18.6% |
| Never smoker (I have never smoked a cigarette) | 550 | 49.4% |
| Non-smoker, vaper (I don’t smoke and have never smoked a cigarette, I only use an e-cigarette/vape) | 64 | 5.7% |

# Supplementary File 8. Comparison of pack attributes among smokers and non-smokers

|  |  | ***Pack 1*** | | ***Pack 2*** | | ***Pack 3*** | | ***Pack 4*** | | ***Pack 5*** | | ***Pack 6*** | |
| --- | --- | --- | --- | --- | --- | --- | --- | --- | --- | --- | --- | --- | --- |
| Attributes |  | *n* | *%* | *n* | *%* | *n* | *%* | *n* | *%* | *n* | *%* | *n* | *%* |
| Most visually appealing overall | Smoker | 261 | 29.5% | 203 | 22.9% | 100 | 11.3% | 76 | 9% | 99 | 11.2% | 147 | 16.6% |
|  | Non-smoker | 322 | 28.9% | 275 | 24.7% | 115 | 10.3% | 115 | 10% | 100 | 9.0% | 187 | 16.8% |
| Least visually appealing overall | Smoker | 190 | 21.4% | 132 | 14.9% | 114 | 12.9% | 97 | 11% | 151 | 17.0% | 202 | 22.8% |
|  | Non-smoker | 209 | 18.8% | 136 | 12.2% | 156 | 14.0% | 129 | 12% | 254 | 22.8% | 230 | 20.6% |
| Highest quality cigarettes | Smoker | 229 | 25.8% | 181 | 20.4% | 128 | 14.4% | 87 | 10% | 113 | 12.8% | 148 | 16.7% |
|  | Non-smoker | 288 | 25.9% | 275 | 24.7% | 138 | 12.4% | 127 | 11% | 116 | 10.4% | 170 | 15.3% |
| Lowest quality cigarettes | Smoker | 142 | 16.0% | 119 | 13.4% | 127 | 14.3% | 132 | 15% | 161 | 18.2% | 205 | 23.1% |
|  | Non-smoker | 174 | 15.6% | 125 | 11.2% | 169 | 15.2% | 169 | 15% | 237 | 21.3% | 240 | 21.5% |
| Most harmful to health | Smoker | 223 | 25.2% | 196 | 22.1% | 108 | 12.2% | 135 | 15% | 81 | 9.1% | 143 | 16.1% |
|  | Non-smoker | 304 | 27.3% | 240 | 21.5% | 122 | 11.0% | 145 | 13% | 113 | 10.1% | 190 | 17.1% |
| Least harmful to health | Smoker | 156 | 17.6% | 92 | 10.4% | 111 | 12.5% | 102 | 12% | 195 | 22.0% | 230 | 26.0% |
|  | Non-smoker | 173 | 15.5% | 117 | 10.5% | 129 | 11.6% | 120 | 11% | 259 | 23.2% | 316 | 28.4% |
| Easiest to quit | Smoker | 209 | 23.6% | 137 | 15.5% | 112 | 12.6% | 96 | 11% | 128 | 14.4% | 204 | 23.0% |
|  | Non-smoker | 259 | 23.2% | 159 | 14.3% | 133 | 11.9% | 133 | 12% | 171 | 15.4% | 259 | 23.2% |
| Hardest to quit | Smoker | 220 | 24.8% | 174 | 19.6% | 89 | 10.0% | 97 | 11% | 136 | 15.3% | 170 | 19.2% |
|  | Non-smoker | 282 | 25.3% | 221 | 19.8% | 120 | 10.8% | 118 | 11% | 172 | 15.4% | 201 | 18.0% |
| I would not smoke this | Smoker | 200 | 22.6% | 161 | 18.2% | 132 | 14.9% | 119 | 13% | 104 | 11.7% | 170 | 19.2% |
|  | Non-smoker | 260 | 23.3% | 188 | 16.9% | 163 | 14.6% | 157 | 14% | 150 | 13.5% | 196 | 17.6% |
| I would smoke this | Smoker | 205 | 23.1% | 144 | 16.3% | 102 | 11.5% | 90 | 10% | 152 | 17.2% | 193 | 21.8% |
|  | Non-smoker | 209 | 18.8% | 204 | 18.3% | 149 | 13.4% | 124 | 11% | 188 | 16.9% | 240 | 21.5% |
| Most effective | Smoker | 192 | 21.7% | 206 | 23.3% | 103 | 11.6% | 122 | 14% | 96 | 10.8% | 167 | 18.8% |
|  | Non-smoker | 233 | 20.9% | 272 | 24.4% | 118 | 10.6% | 154 | 14% | 164 | 14.7% | 173 | 15.5% |
| Least effective | Smoker | 189 | 21.3% | 95 | 10.7% | 97 | 10.9% | 84 | 9% | 198 | 22.3% | 223 | 25.2% |
|  | Non-smoker | 201 | 18.0% | 139 | 12.5% | 131 | 11.8% | 120 | 11% | 229 | 20.6% | 294 | 26.4% |

# Supplementary File 9. Perception of Packs Among Smokers and Non-Smokers

|  | **Question** |  |  | **Pack 1** | | | | | | | |  | **Pack 2** | | | | | | | | | | **Pack 3** | | | | | | | | | | **Pack 4** | | | | | | | | | | **Pack 5** | | | | | | | | | | **Pack 6** | | | | | | | | | |
| --- | --- | --- | --- | --- | --- | --- | --- | --- | --- | --- | --- | --- | --- | --- | --- | --- | --- | --- | --- | --- | --- | --- | --- | --- | --- | --- | --- | --- | --- | --- | --- | --- | --- | --- | --- | --- | --- | --- | --- | --- | --- | --- | --- | --- | --- | --- | --- | --- | --- | --- | --- | --- | --- | --- | --- | --- | --- | --- | --- | --- | --- | --- |
|  |  |  | **SA** | | **A** | | **N** | | **DA** | | **SDA** | | **SA** | | **A** | | **N** | | **DA** | | **SDA** | | **SA** | | **A** | | **N** | | **DA** | | **SDA** | | **SA** | | **A** | | **N** | | **DA** | | **SDA** | | **SA** | | **A** | | **N** | | **DA** | | **SDA** | | **SA** | | **A** | | **N** | | **DA** | | **SDA** | |
|  |  |  | **n** | **%** | **n** | **%** | **n** | **%** | **n** | **%** | **n** | **%** | **n** | **%** | **n** | **%** | **n** | **%** | **n** | **%** | **n** | **%** | **n** | **%** | **n** | **%** | **n** | **%** | **n** | **%** | **n** | **%** | **n** | **%** | **n** | **%** | **n** | **%** | **n** | **%** | **n** | **%** | **n** | **%** | **n** | **%** | **n** | **%** | **n** | **%** | **n** | **%** | **n** | **%** | **n** | **%** | **n** | **%** | **n** | **%** | **n** | **%** |
| 1 | Overall, to what extent do you agree that this pack design is appealing to you (i.e. you like the pack/ pack is attractive/)? | Smoker | *233* | *26.3* | *328* | *37.0* | *165* | *18.6* | *115* | *13.0* | *45* | *5.1* | *192* | 21.7 | *356* | *40.2* | *189* | *21.3* | *111* | *12.5* | *38* | *4.3* | *156* | *17.6* | *310* | *35.0* | *233* | *26.3* | *152* | *17.2* | *35* | *4.0* | *169* | *19.1* | *312* | *35.2* | *205* | *23.1* | *147* | *16.6* | *53* | *6.0* | *200* | *22.6* | *333* | *37.6* | *188* | *21.2* | *123* | *13.9* | *42* | *4.7* | *217* | *24.5* | *327* | *36.9* | *182* | *20.5* | *106* | *12.0* | *54* | *6.1* |
|  |  | Non-smoker | 237 | *21.3* | 363 | *32.6* | 271 | *24.3* | 162 | *14.5* | 81 | *7.3* | 239 | 21.5 | 371 | *33.3* | 270 | *24.2* | 140 | *12.6* | 94 | *8.4* | 152 | *13.6* | 321 | *28.8* | 336 | *30.2* | 198 | *17.8* | 107 | *9.6* | 204 | *18.3* | 339 | *30.4* | 291 | *26.1* | 170 | *15.3* | 110 | *9.9* | 196 | *17.6* | 329 | *29.5* | 269 | *24.1* | 197 | *17.7* | 123 | *11.0* | 229 | *20.6* | 333 | *29.9* | 258 | *23.2* | 163 | *14.6* | 131 | *11.8* |
| 2 | Overall, to what extent do you agree that this pack design encourages you to try smoking or buy the pack? | Smoker | 159 | *17.9* | 319 | *36.0* | 218 | *24.6* | 142 | *16.0* | 48 | *5.4* | 133 | 15.0 | 333 | *37.6* | 236 | *26.6* | 139 | *15.7* | 45 | *5.1* | 122 | *13.8* | 299 | *33.7* | 257 | *29.0* | 166 | *18.7* | 42 | *4.7* | 120 | *13.5* | 295 | *33.3* | 261 | *29.5* | 166 | *18.7* | 44 | *5.0* | 169 | *19.1* | 310 | *35.0* | 233 | *26.3* | 131 | *14.8* | 43 | *4.9* | 161 | *18.2* | 323 | *36.5* | 216 | *24.4* | 142 | *16.0* | 44 | *5.0* |
|  |  | Non-smoker | 129 | *11.6* | 322 | *28.9* | 290 | *26.0* | 239 | *21.5* | 134 | *12.0* | 148 | 13.3 | 290 | *26.0* | 306 | *27.5* | 223 | *20.0* | 147 | *13.2* | 91 | *8.2* | 287 | *25.8* | 340 | *30.5* | 244 | *21.9* | 152 | *13.6* | 117 | *10.5* | 282 | *25.3* | 310 | *27.8* | 245 | *22.0* | 160 | *14.4* | 107 | *9.6* | 298 | *26.8* | 319 | *28.6* | 232 | *20.8* | 158 | *14.2* | 140 | *12.6* | 281 | *25.2* | 283 | *25.4* | 241 | *21.6* | 169 | *15.2* |
| 3 | Overall, to what extent do you agree that you would like to try smoking the cigarettes contained in this pack? | Smoker | 156 | *17.6* | 360 | *40.6* | 213 | *24.0* | 123 | *13.9* | 34 | *3.8* | 144 | 16.3 | 325 | *36.7* | 236 | *26.6* | 136 | *15.3* | 45 | *5.1* | 115 | *13.0* | 304 | *34.3* | 274 | *30.9* | 154 | *17.4* | 39 | *4.4* | 126 | *14.2* | 295 | *33.3* | 265 | *29.9* | 161 | *18.2* | 39 | *4.4* | 139 | *15.7* | 352 | *39.7* | 234 | *26.4* | 125 | *14.1* | 36 | *4.1* | 152 | *17.2* | 341 | *38.5* | 219 | *24.7* | 131 | *14.8* | 43 | *4.9* |
|  |  | Non-smoker | 119 | *10.7* | 295 | *26.5* | 328 | *29.4* | 214 | *19.2* | 158 | *14.2* | 122 | 11.0 | 292 | *26.2* | 291 | *26.1* | 232 | *20.8* | 177 | *15.9* | 95 | *8.5* | 274 | *24.6* | 322 | *28.9* | 277 | *24.9* | 146 | *13.1* | 130 | *11.7* | 254 | *22.8* | 323 | *29.0* | 236 | *21.2* | 171 | *15.4* | 122 | *11.0* | 274 | *24.6* | 325 | *29.2* | 238 | *21.4* | 155 | *13.9* | 148 | *13.3* | 271 | *24.3* | 297 | *26.7* | 214 | *19.2* | 184 | *16.5* |
| 4 | Overall, to what extent do you agree that you would like to be seen with this pack? | Smoker | 131 | *14.8* | 322 | *36.3* | 234 | *26.4* | 143 | *16.1* | 56 | *6.3* | 141 | 15.9 | 299 | *33.7* | 238 | *26.9* | 147 | *16.6* | 61 | *6.9* | 119 | *13.4* | 282 | *31.8* | 266 | *30.0* | 172 | *19.4* | 47 | *5.3* | 113 | *12.8* | 290 | *32.7* | 271 | *30.6* | 155 | *17.5* | 57 | *6.4* | 124 | *14.0* | 330 | *37.2* | 247 | *27.9* | 136 | *15.3* | 49 | *5.5* | 149 | *16.8* | 313 | *35.3* | 222 | *25.1* | 154 | *17.4* | 48 | *5.4* |
|  |  | Non-smoker | 130 | *11.7* | 296 | *26.6* | 296 | *26.6* | 219 | *19.7* | 173 | *15.5* | 142 | 12.7 | 289 | *25.9* | 304 | *27.3* | 204 | *18.3* | 175 | *15.7* | 89 | *8.0* | 276 | *24.8* | 350 | *31.4* | 244 | *21.9* | 155 | *13.9* | 133 | *11.9* | 259 | *23.2* | 322 | *28.9* | 239 | *21.5* | 161 | *14.5* | 122 | *11.0* | 302 | *27.1* | 308 | *27.6* | 231 | *20.7* | 151 | *13.6* | 146 | *13.1* | 277 | *24.9* | 305 | *27.4* | 224 | *20.1* | 162 | *14.5* |
| 5 | Overall, to what extent do you agree that smoking the cigarettes in this pack is harmful to your health? | Smoker | 267 | *30.1* | 368 | *41.5* | 180 | *20.3* | 52 | *5.9* | 19 | *2.1* | 283 | 31.9 | 387 | *43.7* | 149 | *16.8* | 48 | *5.4* | 19 | *2.1* | 214 | *24.2* | 384 | *43.3* | 206 | *23.3* | 68 | *7.7* | 14 | *1.6* | 240 | *27.1* | 391 | *44.1* | 175 | *19.8* | 62 | *7.0* | 18 | *2.0* | 229 | *25.8* | 370 | *41.8* | 200 | *22.6* | 68 | *7.7* | 19 | *2.1* | 251 | *28.3* | 388 | *43.8* | 166 | *18.7* | 62 | *7.0* | 19 | *2.1* |
|  |  | Non-smoker | 444 | *39.9* | 335 | *30.1* | 213 | *19.1* | 73 | *6.6* | 49 | *4.4* | 445 | 39.9 | 354 | *31.8* | 218 | *19.6* | 64 | *5.7* | 33 | *3.0* | 324 | *29.1* | 408 | *36.6* | 255 | *22.9* | 94 | *8.4* | 33 | *3.0* | 388 | *34.8* | 366 | *32.9* | 243 | *21.8* | 83 | *7.5* | 34 | *3.1* | 337 | *30.3* | 400 | *35.9* | 243 | *21.8* | 93 | *8.3* | 41 | *3.7* | 379 | *34.0* | 367 | *32.9* | 228 | *20.5* | 91 | *8.2* | 49 | *4.4* |
| 6 | Overall, to what extent do you agree that the health warnings labels on the front of each of these packs are noticeable? | Smoker | 303 | *34.2* | 363 | *41.0* | 149 | *16.8* | 59 | *6.7* | 12 | *1.4* | 301 | 34.0 | 390 | *44.0* | 155 | *17.5* | 30 | *3.4* | 10 | *1.1* | 216 | *24.4* | 405 | *45.7* | 184 | *20.8* | 59 | *6.7* | 22 | *2.5* | 260 | *29.3* | 387 | *43.7* | 176 | *19.9* | 47 | *5.3* | 16 | *1.8* | 227 | *25.6* | 406 | *45.8* | 182 | *20.5* | 53 | *6.0* | 18 | *2.0* | 288 | *32.5* | 370 | *41.8* | 162 | *18.3* | 49 | *5.5* | 17 | *1.9* |
|  |  | Non-smoker | 403 | *36.2* | 378 | *33.9* | 200 | *18.0* | 96 | *8.6* | 37 | *3.3* | 434 | 39.0 | 381 | *34.2* | 202 | *18.1* | 65 | *5.8* | 32 | *2.9* | 298 | *26.8* | 435 | *39.0* | 250 | *22.4* | 103 | *9.2* | 28 | *2.5* | 375 | *33.7* | 390 | *35.0* | 236 | *21.2* | 77 | *6.9* | 36 | *3.2* | 318 | *28.5* | 396 | *35.5* | 249 | *22.4* | 113 | *10.1* | 38 | *3.4* | 364 | *32.7* | 410 | *36.8* | 212 | *19.0* | 83 | *7.5* | 45 | *4.0* |
| 7 | Overall, to what extent do you agree that the health warnings labels on the front of each of these packs stands out to you/ catches your attention? | Smoker | 280 | *31.6* | 382 | *43.1* | 156 | *17.6* | 51 | *5.8* | 17 | *1.9* | 276 | 31.2 | 375 | *42.3* | 176 | *19.9* | 45 | *5.1* | 14 | *1.6* | 205 | *23.1* | 390 | *44.0* | 208 | *23.5* | 68 | *7.7* | 15 | *1.7* | 250 | *28.2* | 385 | *43.5* | 196 | *22.1* | 45 | *5.1* | 10 | *1.1* | 227 | *25.6* | 388 | *43.8* | 178 | *20.1* | 75 | *8.5* | 18 | *2.0* | 258 | *29.1* | 377 | *42.6* | 177 | *20.0* | 59 | *6.7* | 15 | *1.7* |
|  |  | Non-smoker | 372 | *33.4* | 389 | *34.9* | 225 | *20.2* | 86 | *7.7* | 42 | *3.8* | 426 | 38.2 | 369 | *33.1* | 218 | *19.6* | 69 | *6.2* | 32 | *2.9* | 289 | *25.9* | 387 | *34.7* | 284 | *25.5* | 116 | *10.4* | 38 | *3.4* | 362 | *32.5* | 381 | *34.2* | 248 | *22.3* | 92 | *8.3* | 31 | *2.8* | 290 | *26.0* | 415 | *37.3* | 248 | *22.3* | 117 | *10.5* | 44 | *3.9* | 358 | *32.1* | 384 | *34.5* | 244 | *21.9* | 85 | *7.6* | 43 | *3.9* |
| 8 | Overall, to what extent do you agree that each of these packs make you ‘stop and think about the harmful effects of smoking’ when you look at them? | Smoker | 217 | *24.5* | 388 | *43.8* | 200 | *22.6* | 68 | *7.7* | 13 | *1.5* | 246 | 27.8 | 377 | *42.6* | 183 | *20.7* | 66 | *7.4* | 14 | *1.6* | 189 | *21.3* | 380 | *42.9* | 230 | *26.0* | 75 | *8.5* | 12 | *1.4* | 239 | *27.0* | 379 | *42.8* | 196 | *22.1* | 55 | *6.2* | 17 | *1.9* | 220 | *24.8* | 379 | *42.8* | 192 | *21.7* | 74 | *8.4* | 21 | *2.4* | 240 | *27.1* | 379 | *42.8* | 184 | *20.8* | 66 | *7.4* | 17 | *1.9* |
|  |  | Non-smoker | 349 | *31.3* | 388 | *34.8* | 245 | *22.0* | 97 | *8.7* | 35 | *3.1* | 404 | 36.3 | 364 | *32.7* | 249 | *22.4* | 61 | *5.5* | 36 | *3.2* | 291 | *26.1* | 425 | *38.2* | 263 | *23.6* | 95 | *8.5* | 40 | *3.6* | 362 | *32.5* | 397 | *35.6* | 231 | *20.7* | 85 | *7.6* | 39 | *3.5* | 304 | *27.3* | 382 | *34.3* | 280 | *25.1* | 107 | *9.6* | 41 | *3.7* | 367 | *32.9* | 394 | *35.4* | 215 | *19.3* | 97 | *8.7* | 41 | *3.7* |
| 9 | Overall, to what extent do you agree that the message of the health warnings label on this pack is easy to understand? | Smoker | 295 | *33.3* | 393 | *44.4* | 148 | *16.7* | 41 | *4.6* | 9 | *1.0* | 285 | 32.2 | 419 | *47.3* | 137 | *15.5* | 33 | *3.7* | 12 | *1.4* | 220 | *24.8* | 411 | *46.4* | 184 | *20.8* | 50 | *5.6* | 21 | *2.4* | 257 | *29.0* | 416 | *47.0* | 166 | *18.7* | 32 | *3.6* | 15 | *1.7* | 243 | *27.4* | 404 | *45.6* | 177 | *20.0* | 48 | *5.4* | 14 | *1.6* | 285 | *32.2* | 387 | *43.7* | 160 | *18.1* | 40 | *4.5* | 14 | *1.6* |
|  |  | Non-smoker | 413 | *37.1* | 392 | *35.2* | 207 | *18.6* | 69 | *6.2* | 33 | *3.0* | 414 | 37.2 | 392 | *35.2* | 229 | *20.6* | 53 | *4.8* | 26 | *2.3* | 304 | *27.3* | 421 | *37.8* | 259 | *23.2* | 96 | *8.6* | 34 | *3.1* | 360 | *32.3* | 425 | *38.2* | 230 | *20.6* | 75 | *6.7* | 24 | *2.2* | 328 | *29.4* | 411 | *36.9* | 241 | *21.6* | 99 | *8.9* | 35 | *3.1* | 381 | *34.2* | 393 | *35.3* | 225 | *20.2* | 69 | *6.2* | 46 | *4.1* |

# Supplementary File 10. Ethics Clearance


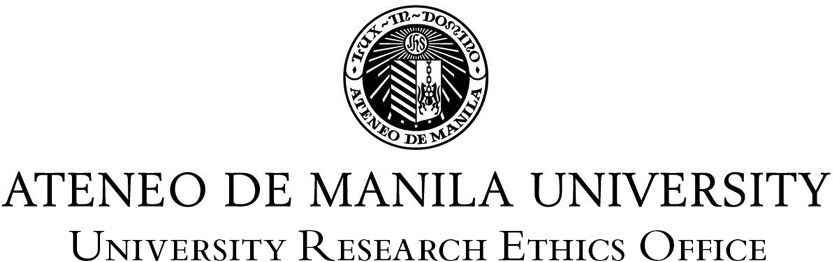


27 July 2022

Ivyrose S. Baysic Ateneo Policy Center School of Government

Ateneo de Manila University Protocol ID: AdMUREC_21_131

Project Title: Promoting the Tobacco Plain Packaging Bill Re: Application for Initial Ethics Approval

Dear Ms. Baysic:

Good day! We are pleased to inform you that your application for initial ethics approval for your project has been reviewed and given clearance to proceed by the University Research Ethics Committee. This approval is based on the application form and relevant materials you submitted dated *19 May 2022*, *with an important addendum sent on 6 and 13 July 2022 .* Your initial ethics approval is valid *until 26 July 2023.*

You are expected to comply with the policies of the Ateneo de Manila University’s Code of

Research Ethics and the Philippine Health Research Ethics Board. Moreover:

- Substantial changes made to your protocol must be reported to and approved by the UREC *prior* to implementation, via AdMUREC Form 5 - Protocol Amendment Form, unless such a change is necessary to avoid immediate harm to the participants. In such a case, the change or amendment must still be reported.
- Any unanticipated problems and/or adverse events that involve risks to participants must be reported to the UREC via AdMUREC Form 8 - Unanticipated Problems Report Form, within one week of the investigator becoming aware of this problem or adverse event.
- If data collection with human participants extend beyond the validity of the initial ethics approval, the proponent must submit AdMUREC Form 6 – Continuing Review Application 45 days prior to the expiration of the initial approval.
- If the project is complete, the proponent must submit AdMUREC Form 11 – Final Report Form within 30 days of completion of the study. Completion indicates that there are no further interactions with and data collection from human participants in the study, and that the data is being handled and/or stored in accordance with the UREC-approved protocol.

Rm 204, 2/F Xavier Hall, Katipunan Avenue, Loyola Heights, 1108 Quezon City, Philippines Tel +63 2 8426 6001 ext 4030, Email [univresearchethics@ateneo.edu](mailto:univresearchethics@ateneo.edu)

- The abovementioned reports must be submitted to the University Research Ethics Office (UREO) and succeeding actions of the researcher must be done after consultation with the UREO or UREC. Kindly contact the UREO for questions or more information.

We wish you success in your research undertaking.


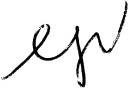
Respectfully yours,

Atty. Eduardo Victor J. Valdez, Ph.D.

Vice-chair

University Research Ethics Committee

Noted by:


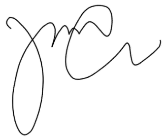


Ronald Allan L. Cruz, Ph.D.

Director

University Research Ethics Office
